# Cross-sectional gut microbiota and serum metabolite differences across clinically defined groups in colorectal cancer

**DOI:** 10.3389/fcimb.2026.1815707

**Published:** 2026-07-10

**Authors:** Zhaodi Jiang, Liqiu Li, Qin Long, Wei Guo, Mingxuan Wang, Xiaoyue Li, Junwei Li, Yongxiang Yi

**Affiliations:** 1 The Second Hospital of Nanjing, Affiliated Hospital to Nanjing University of Chinese Medicine, Nanjing University of Chinese Medicine, Nanjing, China; 2 Taizhou Campus,Zhejiang Cancer Hospital, Taizhou, China; 3 Kunshan Hospital of Chinese Medicine, Affiliated Hospital of Nanjing University of Chinese Medicine, Kunshan, China; 4 Nanjing Drum Tower Hospital Clinical College of Traditional Chinese and Western Medicine, Nanjing University of Chinese Medicine, Nanjing, China

**Keywords:** candidate features, clinical groups, colorectal cancer, gut microbiota, integrated analysis, serum metabolites

## Abstract

Colorectal cancer (CRC) is a prevalent malignancy associated with alterations in the gut microbiota and host metabolic profiles. This cross-sectional study aimed to characterize gut microbiota and serum metabolite differences among healthy controls (HC), patients with non-metastatic colorectal cancer (CRC-nm), and patients with metastatic colorectal cancer (CRC-m). Stool metagenomic sequencing and untargeted serum metabolomics were performed in 107 participants, followed by exploratory differential analyses and internally cross-validated modeling to identify candidate microbial and metabolic features and evaluate their discriminatory performance. Differential analyses identified two CRC-m–enriched species-level features (Enterocloster clostridioformis and Lactobacillus crispatus) and two CRC-m–depleted features (Megamonas rupellensis and Phocaeicola plebeius) across comparisons with both CRC-nm and HC groups. Metabolomic analysis identified eight pathway-mapped metabolites, mainly involved in amino acid–related metabolic pathways. In modeling analyses, metabolite-only models provided the primary discriminatory signal, whereas adding bacterial features did not improve predictive performance. Integrated microbiota–metabolite models showed lower internal performance than metabolite-only models in some comparisons, including CRC-m versus CRC-nm. Overall, these findings suggest that observed discriminatory performance was primarily driven by serum metabolite features rather than additional bacterial features, and highlight candidate microbial and metabolic markers for future validation. Because all CRC-m cases were stage IV and all CRC-nm cases were stages I–III, these results should be interpreted as exploratory cross-sectional group differences that may reflect disease stage, tumor burden, or broader progression-related changes rather than metastasis-specific biology.

## Introduction

Colorectal cancer (CRC) is the third most commonly diagnosed cancer worldwide and the second leading cause of cancer-related mortality, with more than 3 million new cases projected globally by 2040 (Xi and Xu, 2021). Although outcomes for localized disease have improved, distant metastasis remains a major determinant of CRC-related death (Fan et al., 2024). The liver and lungs are the most common sites of metastatic spread in CRC (Biller and Schrag, 2021). Therefore, better characterization of clinical-group-related microbial and metabolic differences in CRC and the identification of candidate features for further validation remain important for improving patient stratification and informing future prognostic research.

The human gut hosts a complex microbial community composed of bacteria, fungi, archaea, and viruses, with approximately 10^13–10^14 microorganisms and more than 1,000 bacterial species (Sender et al., 2016). This gut microbiota forms a symbiotic relationship with the host, and its metagenome encodes 150–300 times more genes than the human genome, leading to its description as the “second human genome” (Qin et al., 2010). Intestinal homeostasis, which reflects the dynamic balance of microbial composition, abundance, and function, is critical for host health, and its disruption is closely linked to CRC and other diseases (O'Hara and Shanahan, 2006). Gut microbiota and their associated products jointly regulate intestinal homeostasis. Beneficial microbial taxa, such as *Faecalibacterium* and *Bifidobacterium*, and microbiota-related products, including short-chain fatty acids and indole derivatives, contribute to the maintenance of epithelial barrier integrity, modulation of immune responses, and stabilization of the commensal microbial community (El Aidy et al., 2013; Martin et al., 2023). By contrast, dysbiosis is more accurately defined as an ecological imbalance characterized by loss of beneficial commensals, reduced microbial diversity, expansion of pathobionts, and altered microbial functions or metabolic outputs, rather than simply the overgrowth of classical enteric pathogens (Petersen and Round, 2014). In this context, altered bile acid pools, trimethylamine-related metabolites, hydrogen sulfide, and pro-inflammatory microbial components such as lipopolysaccharide may contribute to epithelial injury, immune dysregulation, and tumor-promoting inflammation (Rumyantsev et al., 2024). Importantly, lipopolysaccharide is a bacterial structural component rather than a metabolite in the strict sense (Petersen and Round, 2014).

With the rapid development of metagenomics and metabolomics, increasing attention has focused on gut microbiota and their related metabolites as contributors to CRC biology. Previous studies have shown that alterations in gut microbiota are closely associated with CRC initiation and progression (Arthur and Jobin, 2011). For example, *Enterococcus faecalis* and *Streptococcus bovis* may promote CRC development through inflammatory responses (Wang and Huycke, 2015; Deng et al., 2020). In addition, experimental and clinical studies suggest that gut bacteria may be involved in CRC progression and metastatic processes; for example, Escherichia coli has been reported to disrupt the gut vascular barrier and promote bacterial translocation, thereby facilitating CRC liver metastasis (Bertocchi et al., 2021). Gut microbiota and their metabolites can also influence the expression of CRC-related genes (Hullar and Fu, 2014). Isobutyric acid has been reported to be involved in CRC metastatic progression through activation of RACK1 (Chen et al., 2023). Similarly, formate, a metabolite produced by the CRC-associated bacterium *Fusobacterium nucleatum*, has been implicated in CRC progression (Ternes et al., 2022). In mice with CRC liver metastasis, sodium butyrate has been shown to modulate gut microbiota and immune responses (Ma et al., 2020).

Several studies have jointly analyzed metagenomic and metabolomic data in adenoma and CRC and have reported microbe–metabolite alterations relevant to colorectal tumorigenesis (Chen et al., 2022; Coker et al., 2022). These studies support the feasibility of integrated multi-omics analysis for characterizing CRC-associated microbial and metabolic differences. However, studies comparing gut microbiota and serum metabolite profiles across clinically defined CRC groups, particularly cohorts including patients with distant metastasis, remain limited. Recent evidence has linked tumor- or gut-associated microbiota with distant metastasis-related outcomes in CRC, including studies of primary tumor microbiomes and gut microbiota-mediated bile acid metabolism (Parajuli et al., 2025; Zheng et al., 2025). Therefore, cross-sectional differences in gut microbiota and serum metabolite profiles across clinically defined CRC groups remain insufficiently characterized, especially in cohorts including patients with distant metastasis.

In the present study, we performed a cross-sectional integrated analysis of gut microbiota and serum metabolite profiles across three clinically defined groups: healthy controls (HC), patients with non-metastatic colorectal cancer (CRC-nm), and patients with colorectal cancer with distant metastasis (CRC-m). The aim was to characterize cross-sectional microbial and metabolic differences across clinically defined CRC groups, rather than to identify metastasis-specific biomarkers. In the FDR-controlled exploratory workflow, two shared CRC-m-enriched candidate species-level features (Enterocloster clostridioformis and Lactobacillus crispatus), two shared CRC-m-depleted candidate features (Megamonas rupellensis and Phocaeicola plebeius), and eight pathway-mapped metabolites were selected for descriptive interpretation. Because all CRC-m cases were stage IV and all CRC-nm cases were stages I–III, these findings should be interpreted as exploratory cross-sectional group differences that may reflect disease stage, tumor burden, or broader progression-related changes, rather than metastasis-specific biology or validated diagnostic biomarkers.

## Materials and methods

### Participant information

Inclusion criteria were as follows: all CRC patients were confirmed based on the histopathological examination of tumor tissue, with metastatic CRC (CRC-m) defined as the presence of distant metastasis verified by imaging examinations (contrast-enhanced computed tomography [CE-CT] and/or magnetic resonance imaging [MRI], with positron emission tomography-computed tomography [PET-CT] if necessary) interpreted by clinical radiologists in the routine diagnosis and treatment process, non-metastatic CRC (CRC-nm) defined as the absence of evidence of distant metastasis on imaging evaluation prior to sample collection and/or treatment, and healthy controls defined as individuals without a history of CRC or other malignant diseases, without a known history of colorectal adenomas, colorectal polyps, inflammatory bowel disease, or other major gastrointestinal disorders, and with no gastrointestinal symptoms at enrollment. All healthy controls underwent colonoscopic evaluation before inclusion, and no polyps, adenomas, inflammatory lesions, or other colorectal abnormalities were detected. In addition, no abnormalities were identified on routine imaging and clinical examinations.

The exclusion criteria were as follows: (1) concurrent other malignant neoplasms (e.g., tumors at other primary sites or leukemia); (2) a history of receiving any anti-cancer therapy; (3) administration of oral antibiotics, prebiotics, probiotics, or similar agents within 2 months prior to stool sampling; and (4) incomplete medical records.

A total of 107 participants were finally enrolled in the study, including 31 healthy controls, 42 CRC-nm patients, and 34 CRC-m patients. Demographic and clinical data, imaging records, and dietary habits were obtained from hospital electronic medical records and questionnaires (**Supplementary Table 1**). Dietary information was collected only at a coarse level as a general self-reported habitual diet pattern rather than through a validated dietary assessment tool (e.g., food frequency questionnaire or detailed dietary recall). Because all participants were broadly categorized as having a mixed diet, no quantitative dietary variables were available for further stratified or multivariable analysis. All samples were collected at the Second Hospital of Nanjing.

The mean age was 59.94 years in the CRC-m group, 62.18 years in the CRC-nm group, and 55.45 years in the HC group (**Table 1**). This study was approved by the Ethics Committee of the Second Hospital of Nanjing (Approval No. 2022-LS-ky034), and all participants provided written informed consent.

**Table 1:** Clinical and pathological characteristics of the CRC-m, CRC-nm, and HC groups.

Clinical and pathological indexes	CRC-m (n=34)	CRC-nm (n=42)	HC (n=31)	P values CRC-m VS. CRC-nm	P values CRC-m VS. HC
Age (years)	59.94 ± 12.87	62.18 ± 9.51	55.45 ± 8.23	0.268	0.096
Gender				1	0.978
Female	12 (35)	15 (36)	12 (39)		
Male	22 (65)	27 (64)	19 (61)		
BMI	22.8 ± 3.9	24.44± 3.23	22.68 ± 1.29	0.054	0.865
Histology				0.197	NA
adenocarcinoma	32 (94)	42 (100)	NA		
Other	2 (6)		NA		
CEA				0.78	<0.001
<5ng/ml	11 (32)	16 (38)	31 (100)		
>5ng/ml	23 (68)	26 (62)	0 (0)		
Primary tumor sites				0.096	NA
Right colon	7 (21)	10 (24)	NA		
Left colon	15 (44)	9 (21)	NA		
Rectum	12 (35)	23 (55)	NA		
Tumor stage					
I	0 (0)	5 (12)	NA		
II	0 (0)	20 (48)	NA		
III	0 (0)	17 (40)	NA		
IV	34 (100)	0 (0)	NA		

### Sample collection

Samples were collected from October 2022 to May 2023. Stool and peripheral blood samples were collected from each participant. Stool samples were transported on ice, snap-frozen in liquid nitrogen, and stored at -80 °C. Serum was separated from peripheral blood by centrifugation and stored at -80 °C.

### Microbial DNA extraction, metagenomic sequencing, and data processing

DNA extraction from stool specimens was conducted using the QIAamp 96 Power Fecal QIAcube HT system (Qiagen, Germany). The DNA was then quantified and assessed for integrity using a NanoDrop 2000 spectrophotometer (Thermo Fisher) and agarose gel electrophoresis. The libraries were prepared using the TruSeq Nano DNA LT Sample Preparation Kit (Illumina, San Diego, CA, USA). DNA fragmentation was performed with the Covaris S220 Focused-Ultrasonicators (Covaris, USA) under settings designed for optimal fragment sizes (350–500 bp). DNA purification was carried out using Agencourt AMPure XP beads (Beckman Coulter, USA). Sequencing was carried out on the Illumina NovaSeq 6000 platform, using 150 bp paired-end reads. This high-throughput platform supports large-scale sequencing and provides sufficient coverage for metagenomic applications. Sequencing generated 68.57–175.67 million raw paired-end reads per sample (mean 104.53 million). After quality control, 68.51–175.53 million clean reads per sample were retained (mean 104.44 million), corresponding to 10.26–26.16 Gb clean bases per sample (mean 15.63 Gb). Detailed per-sample raw-read, clean-read, clean-base, and dehosted-read statistics are provided in **Supplementary Table 2**. After host-read removal, the retained dehosted reads ranged from 20.02 to 175.03 million per sample (mean 103.11 million; median 100.72 million). Although dehosted read depth varied across samples, all samples retained more than 20 million dehosted reads after host-read removal. Therefore, no sample was excluded due to insufficient retained microbial reads, and the retained depth was considered sufficient for the present group-level metagenomic taxonomic and gene-catalog-based analyses. After sequencing, raw reads were processed using fastp (v0.20.1) for adapter trimming and quality filtering, and host-derived reads were removed by alignment to the human reference genome GRCh38.p13 using bbmap (v38.93-0). Metagenomic assembly was performed with MEGAHIT (v1.1.2) (Li et al., 2015), and open reading frame (ORF) prediction was performed using Prodigal (v2.6.3) (Hyatt et al., 2010). The non-redundant gene catalog was constructed with CD-HIT (v4.6.7) (Li et al., 2001), and functional annotation was carried out by aligning the sequences to the National Center for Biotechnology Information non-redundant protein database (NCBI NR), Kyoto Encyclopedia of Genes and Genomes (KEGG), Swiss-Prot, and Clusters of Orthologous Groups (COG) databases using DIAMOND (v0.9.7). Taxonomic profiles were derived by annotating the representative sequences of the non-redundant gene catalog against the taxonomy database associated with the NCBI NR database. For each sample, gene abundance values were obtained from the non-redundant gene abundance table generated in the metagenomic workflow, and species-level abundance was calculated by summing the abundances of all genes annotated to the same species. Because taxonomic annotation was based on reference database labels, some entries retained catalog- or genome-style identifiers (e.g., Akkermansia muciniphila CAG 154). These entries were preserved as separate annotated abundance features in the pipeline output; however, they should not be interpreted as independently named biological species, formally resolved strains, or separate taxonomic entities comparable to conventionally named species. Abundance profiles were then generated at the domain, kingdom, phylum, class, order, family, genus, and species levels.

### Liquid chromatography–mass spectrometry untargeted metabolomics analysis

Serum metabolites were extracted for untargeted liquid chromatography-mass spectrometry (LC-MS) analysis as follows. Briefly, 150 μL of each serum sample was transferred to a 1.5-mL microcentrifuge tube and mixed with 600 μL of pre-cooled methanol-acetonitrile (2:1, v/v) containing L-2-chlorophenylalanine (2 μg/mL) as an internal standard. Samples were vortexed for 1 min, sonicated in an ice-water bath for 10 min, and incubated at -40 °C for 2 h to promote protein precipitation. The extracts were then centrifuged at 14,300 × g for 10 min at 4 °C, and 150 μL of the supernatant was passed through a 0.22-μm organic-phase membrane filter into LC vials for analysis. A pooled quality-control (QC) sample was prepared by mixing equal aliquots of all sample extracts.

LC-MS analysis was performed on an ACQUITY UPLC I-Class Plus system coupled to a Q Exactive high-resolution mass spectrometer equipped with an electrospray ionization (ESI) source. Chromatographic separation was achieved on an ACQUITY UPLC HSS T3 column (100 mm × 2.1 mm, 1.8 μm) maintained at 45 °C. The mobile phases were water containing 0.1% formic acid (A) and acetonitrile (B), delivered at 0.35 mL/min with an injection volume of 3 μL. The gradient was as follows: 0–2 min, 5% B; 2–4 min, 5-30% B; 4–8 min, 30-50% B; 8–10 min, 50-80% B; 10–14 min, 80-100% B; 14–15 min, 100% B; and 15.1–16 min, re-equilibration at 5% B. Mass spectra were acquired in both positive- and negative-ion modes under the following settings: spray voltage, 3.8 kV (positive) and -3.0 kV (negative); capillary temperature, 320 °C; auxiliary gas heater temperature, 350 °C; sheath gas, 35 arb; auxiliary gas, 8 arb; S-lens RF level, 50; scan range, m/z 100-1200; full mass spectrometry (MS) resolution, 70,000; tandem mass spectrometry (MS/MS) resolution, 17,500; stepped normalized collision energy, 10/20/40.

QC samples were injected throughout the analytical sequence to monitor analytical stability. Study-sample injection order was randomized, and pooled QC samples were injected at the beginning and end of the analytical sequence and after every 10 study samples. A solvent blank was included during acquisition; however, blank raw data were not retained in the exported project record and were not used for background-peak or contaminant removal.

Raw LC-MS data were processed in Progenesis QI v3.0, including baseline filtering, peak picking, integration, retention-time correction, peak alignment, and Progenesis QI default signal normalization. The Progenesis QI-normalized and log10-transformed LC-MS matrix was used as the pre-correction matrix shown in **Supplementary Figure 5C**, before QC-based LOESS signal-drift correction, statTarget batch correction, and missing-value imputation. The default Progenesis QI normalization was applied before QC-based LOESS signal-drift correction, statTarget batch correction, and missing-value imputation. L-2-chlorophenylalanine was not used for LC-MS signal normalization but was used as an internal standard to monitor sample-preparation and instrumental stability. QC-based signal-drift correction was performed using the LOESS algorithm based on pooled QC samples, followed by statTarget-based batch correction, with LC-MS and GC-MS data matrices corrected separately. Metabolite annotation was based on accurate mass, MS/MS fragmentation, isotope distribution, and, when available, retention-time information, by matching against the Human Metabolome Database (HMDB), LipidMaps, METLIN, and the EMDB 2.0 in-house library. Precursor and product ion tolerances were set to 5 ppm/10 ppm and 10 ppm/20 ppm for the in-house library and METLIN searches, respectively. Features with >50% missing values within a group were removed, remaining missing values in the LC-MS matrix were imputed using the K-nearest-neighbor (KNN) method, and ion peaks with a relative standard deviation (RSD) >30% across quality-control (QC) samples were excluded before downstream analysis. The LC-MS matrix shown in **Supplementary Figure 5D** represents the same retained feature set after QC-based LOESS correction, statTarget batch correction, and KNN-based missing-value imputation. The dense low-intensity component around log10 values of approximately −6 to −8 in **Supplementary Figure 5D** mainly represented small positive values generated during KNN-based imputation for features with original signals below the detection limit or absent in some samples, and these imputed values were retained for downstream differential metabolite analysis. Putative annotations with a score <36/80 were discarded before merging the positive- and negative-ion data matrices for downstream statistical analysis (Dunn et al., 2011; Sarafian et al., 2014). Accordingly, the reported LC-MS metabolites should be regarded as putatively annotated compounds rather than fully confirmed metabolite identities. Overall analytical stability was evaluated using pooled QC-based quality-control plots, sample-level metabolite intensity distributions before and after correction, internal-standard stability assessment, and feature-level QC filtering; platform-specific QC assessments are provided in **Supplementary Figure 4**, sample-level intensity distributions in **Supplementary Figure 5**, and feature-level QC RSD values for the eight pathway-mapped metabolites and internal-standard signals in **Supplementary Table 9**. After correction, the internal-standard QC RSD values were 6.28% in negative-ion mode and 2.10% in positive-ion mode, supporting acceptable internal-standard stability across QC injections.

### Gas chromatography–mass spectrometry analysis of metabolic profiles

For GC-MS analysis, 150 μL of serum was mixed with 600 μL methanol-acetonitrile (2:1, v/v) containing L-2-chlorophenylalanine (2 μg/mL), vortexed for 1 min, sonicated in an ice-water bath for 10 min, and incubated at -40 °C for 30 min. After centrifugation at 14,300 × g for 10 min at 4 °C, 150 μL of the supernatant was transferred to a glass derivatization vial and evaporated to dryness in a refrigerated centrifugal concentrator. For derivatization, 80 μL methoxyamine hydrochloride in pyridine (15 mg/mL) was added and the sample was reacted at 37 °C for 60 min for oximation. Subsequently, 50 μL N,O-bis(trimethylsilyl)trifluoroacetamide (BSTFA) and 20 μL n-hexane were added together with 10 μL of a mixed internal-standard solution (C8/C9/C10/C12/C14/C16/C18/C20/C22/C24 prepared in chloroform), followed by derivatization at 70 °C for 60 min. The samples were then allowed to stand at room temperature for 30 min prior to GC-MS analysis. A pooled QC sample, prepared by combining equal volumes of all extracts, was injected regularly during the run (typically after every 10 study samples).

GC-MS analysis was performed on an Agilent 7890B-5977B system equipped with a DB-5MS capillary column (30 m × 0.25 mm × 0.25 μm). High-purity helium (>99.999%) was used as the carrier gas at 1.0 mL/min. The injector temperature was 260 °C, the injection volume was 1 μL, and samples were introduced in splitless mode with a solvent delay of 6.2 min. The oven temperature program was: 60 °C for 0.5 min; ramp to 125 °C at 8 °C/min; ramp to 210 °C at 8 °C/min; ramp to 270 °C at 15 °C/min; and ramp to 305 °C at 20 °C/min, followed by a 5-min hold. Mass spectrometric detection was performed using electron-impact ionization at 70 eV, with an ion source temperature of 230 °C, quadrupole temperature of 150 °C, full-scan acquisition mode, and a scan range of m/z 50-500. Raw data files were converted to analysis base file (ABF) format and processed in MS-DIAL for peak detection, MS2Dec deconvolution, retention-time alignment, filtering, and missing-value interpolation. Putative metabolite annotation was achieved by matching retention behavior and EI mass spectra against the LUG (Luming untargeted GC-MS) library. Internal-standard peaks and known artifact peaks (including noise, column bleed, and derivatization-reagent peaks) were removed. Features with >50% missing values within a group were excluded, remaining missing values were replaced with one-half of the minimum detected value, and peak intensities were normalized by retention-time-segmented internal-standard correction using internal standards with RSD <0.1 across all samples. After redundancy removal and peak merging, only metabolites with a total annotation score ≥70/100 were retained for downstream analysis. Thus, for GC-MS, internal standards were used not only experimentally during derivatization but also analytically during data normalization. Overall analytical stability was further evaluated using pooled QC-based quality-control plots. Platform-specific QC assessments are provided in **Supplementary Figure 3**, and sample-level GC-MS intensity distributions before and after correction are shown in **Supplementary Figures 5A, B**. Feature-level QC stability was further evaluated for the eight pathway-mapped metabolites carried forward into the main results, and the corresponding QC RSD values are provided in **Supplementary Table 9**. After correction, the QC RSD values of these eight metabolites ranged from 1.45% to 3.64%, indicating acceptable reproducibility across QC injections. The corrected QC RSD values were 1.45% for 2-amino-3-methylpentanoic acid, 2.99% for L-methionine, 2.50% for L-phenylalanine, 2.36% for L-serine, 2.06% for L-threonine, 3.33% for L-tyrosine, 1.63% for L-valine, and 3.64% for sarcosine.

### Statistical analysis

Statistical analyses were performed using R software (version 4.2.2) unless otherwise specified. Continuous clinical variables in **Table 1**, including age and body mass index (BMI), were compared between groups using Welch’s t-test. Categorical clinical variables, including sex, carcinoembryonic antigen (CEA) category, histology, and primary tumor site, were compared using the chi-square test or Fisher’s exact test, as appropriate.

Alpha diversity indices (Shannon and Simpson) were compared among the three groups using the Kruskal–Wallis test. Beta diversity was assessed by principal coordinates analysis (PCoA) based on weighted UniFrac distance, and overall between-group differences were evaluated using permutational multivariate analysis of variance (PERMANOVA). The resulting weighted UniFrac PERMANOVA statistics (F = 2.57, R^2^ = 0.047, p = 0.001) were recoverable from the retained result file. To further assess whether group differences could be influenced by unequal within-group dispersion, a permutational analysis of multivariate dispersions (PERMDISP) was additionally performed at the species level using Bray-Curtis distance with 999 permutations. Because this PERMDISP analysis was based on Bray-Curtis rather than the same weighted UniFrac distance matrix used for the main PERMANOVA, it was interpreted as a complementary dispersion assessment rather than a direct validation of the weighted UniFrac PERMANOVA result.

For microbial taxonomic comparisons, pairwise differences between CRC-m and CRC-nm and between CRC-m and HC were assessed using the Wilcoxon rank-sum test. The taxonomic composition plots are descriptive stacked bar plots of mean relative abundance and were used to visualize overall compositional patterns across groups rather than for formal statistical inference. Differential species-level microbial features were screened using LEfSe on the stool metagenomic species-level relative abundance matrix after retaining only unambiguously annotated species with a mean relative abundance >0.0001 and prevalence >10% across samples. Linear discriminant analysis effect size (LEfSe) outputs were reported as exploratory candidate species-level microbial features when the Benjamini–Hochberg false discovery rate (FDR)-adjusted p value, calculated across all tested species-level features within each pairwise comparison before feature selection, was <0.10 and the linear discriminant analysis (LDA) score was >2.5. For each pairwise comparison, false discovery rate correction was calculated across all p values generated from the full set of retained species-level features passing the predefined abundance, prevalence, and annotation filters. Complete feature-level outputs for all species-level features entering each pairwise LEfSe comparison, including features not meeting the exploratory reporting criteria, group direction, nominal p values, Benjamini–Hochberg FDR-adjusted p values, and LDA scores, are provided in **Supplementary Table 4** to support transparent interpretation of the exploratory LEfSe screening results. For the comparison of selected key taxa across the three groups, the Kruskal–Wallis test was used, followed by Dunn’s *post hoc* test with Bonferroni correction.

For metabolomics analysis, LC-MS and GC-MS data were analyzed using supervised discriminant analysis in the platform workflow, and partial least squares discriminant analysis (PLS-DA) score plots were used for visualization. Model overfitting was evaluated by 7-fold cross-validation and 200 permutation tests. Additional scaling settings, formal normality testing, exact variance assumptions used in the platform t-test workflow, and some other platform-level parameters for the supervised metabolomics workflow were not explicitly retained in the exported records and therefore could not be fully reconstructed in the present study. Variable importance in projection (VIP) values were derived from the supervised discriminant analysis models implemented in the platform workflow. Differential metabolites between groups were initially screened using the following criteria: VIP > 1, adjusted p < 0.05, and fold change > 1.2 or < 0.83. Adjusted p values were calculated using the Benjamini–Hochberg false discovery rate procedure across the full set of tested metabolite features within each comparison before hit selection. Complete comparison-specific metabolite-level statistical outputs, including metabolite annotation, comparison group, fold change, nominal p value, Benjamini–Hochberg FDR-adjusted p value, VIP value, direction of change, and whether the metabolite met the predefined differential-metabolite criteria, are provided in **Supplementary Tables 5, 6**. Metabolites shared by the CRC-m vs. CRC-nm and CRC-m vs. HC comparisons were used to define a conservative shared metabolite set identified in comparisons involving the CRC-m group for downstream exploratory pathway-oriented analysis. This selection strategy was intended to identify metabolites that consistently differed in the CRC-m group relative to both comparison groups, rather than metabolites specific to metastatic dissemination. Because all CRC-m cases were stage IV and all CRC-nm cases were stages I–III, the CRC-m vs. CRC-nm comparison should be interpreted as a stage- and progression-confounded within-CRC contrast, whereas the CRC-m vs. HC comparison represents a broader disease-vs-healthy contrast. Therefore, the intersected metabolite set was used only as a descriptive input for pathway-level summarization and was not interpreted as evidence of metastasis-specific metabolic biology. For the selected pathway-mapped metabolites, pairwise comparisons between CRC-m and CRC-nm and between CRC-m and HC were performed using unpaired Student’s t-tests on log10-transformed data. Metabolite set enrichment analysis was performed using the Enrichment Analysis module of the MetaboAnalyst 6.0 web server (https://www.metaboanalyst.ca). The list of 42 shared metabolites identified in comparisons involving the CRC-m group was used as input, and compound identifiers were mapped to the KEGG human metabolic pathway library (81 pathways; updated November 2025). Over-representation analysis (ORA) based on the hypergeometric test was applied to evaluate pathway enrichment, using the full set of annotated metabolites detected in this study as the background. P values were adjusted for multiple testing using the Benjamini–Hochberg false discovery rate (FDR) procedure at the pathway level. Pathways with FDR-adjusted P < 0.05 were considered statistically significant, whereas pathways with 0.05 ≤ FDR < 0.10 were considered to show borderline enrichment. The pathway enrichment results retained for reporting, together with enrichment statistics and adjusted P values, are provided in **Supplementary Table 7**.

No arbitrary cutoff based on a fixed number of pathways was applied. Instead, all pathways meeting the predefined statistical criteria were retained. For downstream interpretation, we focused on pathways with the strongest statistical support (FDR < 0.10) and clear biological relevance to amino acid and one-carbon metabolism.

For the exploratory machine-learning analysis, pairwise CRC-m group comparisons were evaluated using three feature sets: bacteria-only features, metabolite-only features, and integrated microbiota–metabolite features. In each comparison, CRC-m samples were assigned to the case group, whereas the corresponding comparator group was assigned to the control group. For the CRC-m versus CRC-nm comparison, CRC-nm samples served as controls; for the CRC-m versus HC comparison, HC samples served as controls. Predictor matrices were constructed after removing sample identifiers and group-label columns, and all remaining variables were converted to numeric values before model fitting.

To reduce optimism and avoid information leakage, model development and evaluation were performed using an outer five-fold stratified cross-validation framework with a fixed random seed. In each outer iteration, four folds were used as the training set and the remaining fold was held out as the validation set. The folds were stratified according to the case-control label to preserve class distribution across training and validation splits. All feature filtering, model tuning, penalized model fitting, and model-based feature selection were performed strictly within the outer training folds. The held-out validation fold was used only for performance evaluation.

For bacteria-only models, species-level bacterial abundance features were used as the initial bacterial feature matrix. Within each outer training fold, bacterial features were filtered using prevalence >10% and mean relative abundance >0.01%, and the resulting training-fold-derived feature set was then applied to the corresponding validation fold. For metabolite-only models, detected serum metabolite features were entered into the models without additional univariate or differential-metabolite prescreening before penalized regression. For integrated microbiota–metabolite models, the training-fold-filtered bacterial feature matrix and the serum metabolite feature matrix were combined within each outer fold before penalized model fitting.

For each feature set, two penalized logistic-regression models were evaluated using the glmnet R package with binomial family: least absolute shrinkage and selection operator (LASSO) logistic regression and elastic net logistic regression. For LASSO logistic regression, the mixing parameter alpha was fixed at 1. The regularization parameter lambda was selected within each outer training set using five-fold internal cross-validation implemented by cv.glmnet, with receiver operating characteristic area under the curve as the tuning criterion. The lambda value corresponding to the best internal cross-validation performance (lambda.min) was then used to fit the final LASSO model on the full outer training set.

For elastic net logistic regression, both alpha and lambda were tuned within each outer training fold, with alpha evaluated from 0.1 to 0.9 and lambda selected for each alpha using five-fold internal cross-validation with ROC-AUC as the tuning criterion. Candidate alpha values ranged from 0.1 to 0.9 in increments of 0.1. For each candidate alpha value, five-fold internal cross-validation was performed using cv.glmnet with receiver operating characteristic area under the curve as the tuning criterion. The alpha value with the highest internal cross-validation performance was selected, and the corresponding lambda.min was used to fit the final elastic net model on the full outer training set.

The final fold-specific models were applied to the corresponding held-out validation folds to generate predicted probabilities. Predicted probabilities were converted into binary class predictions using a fixed probability threshold of 0.50. Model performance was calculated separately in each outer validation fold and included the area under the receiver operating characteristic curve (ROC-AUC), the area under the precision-recall curve (PR-AUC), sensitivity, specificity, positive predictive value (PPV), negative predictive value (NPV), and F1-score. The mean values across the five outer folds were reported as cross-validated internal performance estimates. When a threshold-dependent metric was undefined in a validation fold because no positive or no negative predictions were made, the corresponding fold was excluded from the calculation of the mean and standard deviation for that metric. For visualization, receiver operating characteristic curves were generated separately for each outer validation fold.

To assess model interpretability and feature-selection stability, non-zero coefficients were extracted from the final fold-specific LASSO and elastic net models. Features with non-zero coefficients were considered selected features in the corresponding outer fold. For each model type and feature set, feature-selection results were summarized across the five outer folds by reporting selection frequency, mean non-zero coefficient, and coefficient direction for each selected feature. Because feature selection was repeated independently within each outer training fold, these aggregated selected-feature summaries were interpreted as cross-validation-dependent candidate predictors rather than as a single fixed diagnostic marker panel. All machine-learning analyses were regarded as exploratory internal validation analyses rather than evidence of clinically established diagnostic performance, because no independent external validation cohort was available and CRC-m and CRC-nm were completely confounded by disease stage.

*Post hoc* selected-feature exploratory within-group Spearman correlation analyses were performed separately in the HC, CRC-nm, and CRC-m groups to assess associations between the four retained candidate species-level microbial features, including both CRC-m-enriched features (Enterocloster clostridioformis and Lactobacillus crispatus) and CRC-m-depleted features (Megamonas rupellensis and Phocaeicola plebeius), and the eight pathway-mapped metabolites. Similar *post hoc* selected-feature exploratory correlation analyses were also performed between these candidate microbial features and the systemic inflammatory indices. Correlation coefficients were calculated using Spearman’s rank correlation. P values were adjusted using the Benjamini–Hochberg false discovery rate procedure across the tested metabolite correlations for each microbial species within each group. Adjusted p values <0.05 were considered statistically significant, whereas adjusted p values between 0.05 and 0.10 were interpreted as borderline exploratory associations. The correlation analyses were conducted within each clinical group to reduce the possibility that apparent associations were driven primarily by between-group separation.

## Results

### Characteristics of participants

A total of 107 participants were included in this study. All CRC patients were pathologically confirmed. Additionally, patients in the CRC-m group met the corresponding radiological diagnostic criteria, with double or even multiple primary malignancies excluded. The HC group consisted of individuals who underwent routine physical examinations, had no history of CRC or other malignant diseases, and underwent colonoscopic evaluation before inclusion, with no colorectal polyps, adenomas, inflammatory lesions, or other colorectal abnormalities detected. Metagenomic sequencing was performed on stool samples, and untargeted LC-MS and GC-MS analyses were performed on serum samples. Baseline characteristics, including age, sex, and body mass index (BMI), were collected for all participants. For CRC-nm and CRC-m patients, additional clinical information, including histology and primary tumor site, was collected (**Table 1**).

### Gut microbiota structural changes and compositional differences across HC, CRC-nm, and CRC-m groups

**Figure 1:**
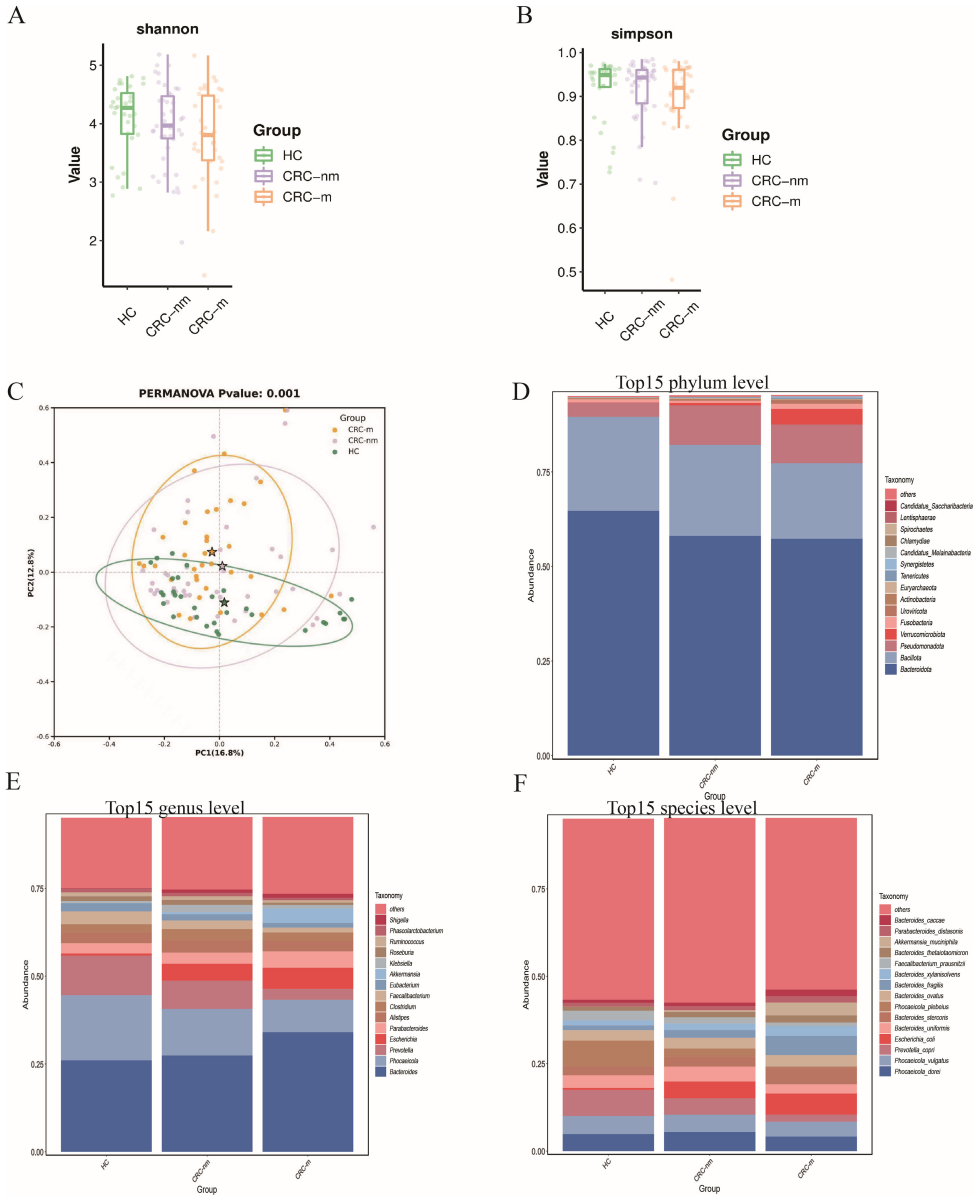
Metagenomic changes and data profiles. **(A, B)** Alpha diversity at the species level was estimated using the Shannon index **(A)** and Simpson index **(B)** in healthy controls (HC), non-metastatic colorectal cancer (CRC-nm), and metastatic colorectal cancer (CRC-m). **(C)** PCoA score plot based on weighted UniFrac distance. Group centroids (stars), representing the average ordination position of each group, were added to facilitate visualization of group-level clustering patterns. The three groups showed an exploratory global difference in gut microbiota composition, with substantial overlap in the ordination space (PERMANOVA, F = 2.57, R^2^ = 0.047, p = 0.001). A complementary species-level PERMDISP analysis based on Bray-Curtis distance showed significant heterogeneity of within-group dispersion among groups (F = 3.9824, p = 0.0210), suggesting that the PERMANOVA result should be interpreted cautiously and not as definitive evidence of distinct group clustering. **(D–F)** Relative abundance of the top 15 taxa at the phylum **(D)**, genus **(E)**, and species **(F)** levels.

No significant differences in alpha diversity at the species level were observed among the three groups (**Figures 1A, B**). To evaluate between-group differences, weighted UniFrac principal coordinates analysis (PCoA) was performed to assess beta diversity. To facilitate visualization of group-level clustering patterns, group centroids representing the average ordination position of each group were added to the PCoA plot. Weighted UniFrac PCoA suggested an exploratory global difference in gut microbiota composition across the three groups, although the ordination showed substantial overlap. PERMANOVA indicated a significant overall group effect (F = 2.57, R^2^ = 0.047, p = 0.001). A complementary species-level PERMDISP analysis based on Bray-Curtis distance showed significant heterogeneity of within-group dispersion among the three groups (F = 3.9824, p = 0.0210). Pairwise PERMDISP comparisons indicated that dispersion differed between CRC-m and HC (BH-adjusted p = 0.0090) and between CRC-nm and HC (BH-adjusted p = 0.0315), but not between CRC-m and CRC-nm (BH-adjusted p = 0.7790). Therefore, the beta-diversity result should be interpreted cautiously, as the observed group effect may partly reflect differences in within-group dispersion rather than only differences in group centroids (**Figure 1C**).

We further characterized gut microbiota composition in the three groups (**Supplementary Table 3**). At the phylum level, Bacteroidota (formerly Bacteroidetes), Bacillota (formerly Firmicutes), Pseudomonadota (formerly Proteobacteria), and Verrucomicrobiota (formerly Verrucomicrobia) were the dominant taxa, accounting for >90% of sequences across samples (**Figure 1D**). At the genus level, Bacteroides and Phocaeicola were among the dominant genera across all three groups (**Figure 1E**). The stacked bar plot of mean relative abundance suggested compositional differences among groups: compared with the CRC-m group, the HC and CRC-nm groups appeared to show relatively lower representation of Bacteroides and relatively higher representation of Phocaeicola. In addition, the mean relative abundance of Prevotella showed a decreasing pattern across the three groups (HC, 11.28%; CRC-nm, 8.05%; CRC-m, 3.12%). Because these observations are based on relative abundance data, they should be interpreted as descriptive compositional shifts rather than absolute quantitative changes. At the species level, Prevotella copri (7.47%), Phocaeicola plebeius (7.37%), and Phocaeicola vulgatus (5.18%) were most abundant in the HC group. In the CRC-nm group, the most abundant species were Phocaeicola dorei (5.44%), Phocaeicola vulgatus (4.95%), and Escherichia coli (4.77%). In the CRC-m group, Escherichia coli (5.99%), Bacteroides fragilis (5.46%), and Bacteroides stercoris (4.62%) were most prevalent (**Figure 1F**).

### Exploratory FDR-controlled LEfSe screening of CRC-m-enriched and CRC-m-depleted candidate species-level microbial features across CRC-m and comparator groups

To screen exploratory candidate species-level microbial features differing between the CRC-m group and comparator groups, including both CRC-m-enriched and CRC-m-depleted features, LEfSe analysis was performed using the stool metagenomic species-level relative abundance matrix after filtering for unambiguously annotated species with a mean relative abundance >0.0001 and prevalence >10%. Within each pairwise comparison, FDR correction was applied across the full set of retained tested species, and features with FDR-adjusted p <0.10 and LDA score >2.5 were retained as exploratory candidate features. In the CRC-m vs. CRC-nm comparison, Enterocloster clostridioformis and Lactobacillus crispatus showed higher relative abundance in the CRC-m group and were interpreted as CRC-m-enriched exploratory candidate species-level features, whereas Megamonas rupellensis and Phocaeicola plebeius showed higher relative abundance in the CRC-nm group and were therefore interpreted as CRC-m-depleted candidate species-level features in this contrast. Notably, *Megamonas rupellensis* met the conventional FDR <0.05 threshold in this comparison. In the CRC-m vs. HC comparison, multiple candidate species-level features met the exploratory reporting criteria, including CRC-m-enriched candidate features such as *Enterocloster clostridioformis* and *Lactobacillus crispatus*, as well as CRC-m-depleted candidate features. Among the CRC-m-depleted taxa, *Megamonas rupellensis* was again enriched in the comparator group and met the conventional FDR <0.05 threshold, indicating that it represented a statistically stronger shared CRC-m-depleted feature across both CRC-m comparisons. Complete feature-level outputs for all species-level features entering each pairwise LEfSe comparison, including features not meeting the exploratory reporting criteria, are provided in **Supplementary Table 4**.

**Figure 2:**
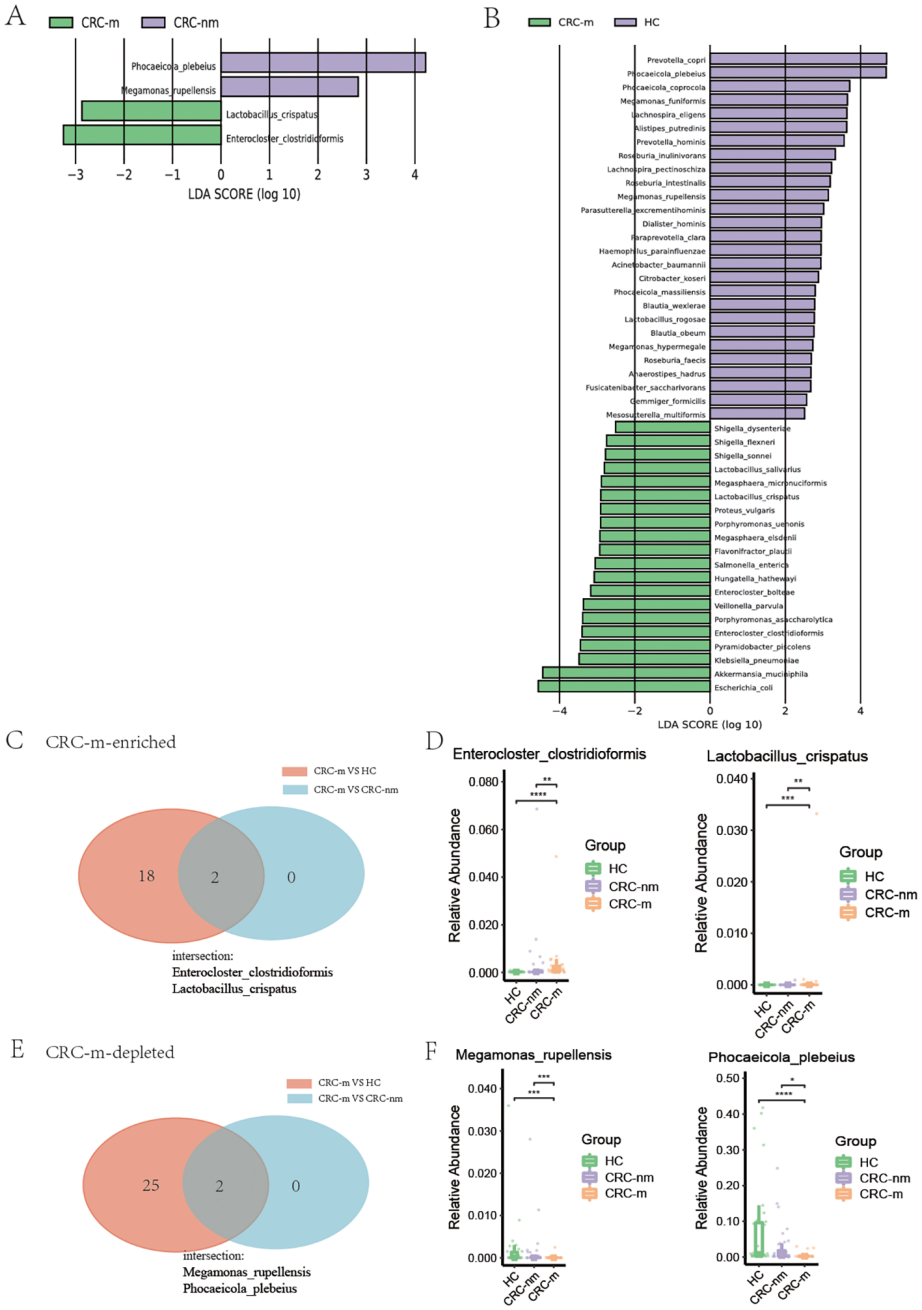
Exploratory FDR-controlled LEfSe screening of CRC-m-enriched and CRC-m-depleted candidate species-level microbial features across CRC-m and comparator groups. **(A, B)** Candidate species-level microbial features retained by LEfSe in CRC-m vs. CRC-nm **(A)** and CRC-m vs. HC **(B)** after filtering for unambiguously annotated species with a mean relative abundance >0.0001 and prevalence >10%. Features shown in the figure met the prespecified exploratory criteria of Benjamini–Hochberg FDR-adjusted p <0.10, calculated across all tested species-level features within each comparison, and LDA score >2.5; these features should be interpreted as candidate discriminant species-level features rather than definitive significant taxa. The complete tested feature-level outputs are provided in **Supplementary Table 4**. **(C)** Overlap of CRC-m-enriched candidate species-level features across the two pairwise comparisons, showing *Enterocloster clostridioformis* and *Lactobacillus crispatus* as shared enriched features. **(D)** Relative abundance distributions of *Enterocloster clostridioformis* and *Lactobacillus crispatus*. **(E)** Overlap of CRC-m-depleted candidate species-level features across the two pairwise comparisons, identifying *Megamonas rupellensis* and *Phocaeicola plebeius* as shared depleted features. **(F)** Relative abundance distributions of *Megamonas rupellensis* and *Phocaeicola plebeius* across HC, CRC-nm, and CRC-m groups. *p < 0.05, **p < 0.01, ***p < 0.001, ****p < 0.0001.

Among the candidate species-level features, Enterocloster clostridioformis and Lactobacillus crispatus were identified as shared CRC-m-enriched exploratory candidate features across both pairwise contrasts (**Figure 2C**). In the clinically relevant CRC-m vs. CRC-nm comparison, these two species met the exploratory FDR < 0.10 threshold but did not reach the conventional FDR < 0.05 threshold, supporting their interpretation as exploratory candidate features. Shared CRC-m-depleted candidate features were also evaluated to characterize taxa showing consistent changes in the opposite direction (**Figure 2E**). Notably, Megamonas rupellensis represented a statistically stronger shared feature meeting the conventional FDR < 0.05 threshold across both contrasts, whereas Phocaeicola plebeius passed the exploratory FDR < 0.10 threshold. Therefore, both the shared CRC-m-enriched features (Enterocloster clostridioformis and Lactobacillus crispatus, **Figure 2D**) and shared CRC-m-depleted features (Megamonas rupellensis and Phocaeicola plebeius, **Figure 2F**) were retained for downstream descriptive visualization. Because the CRC-m and CRC-nm groups differed by disease stage, these candidate species-level differences were interpreted as cross-sectional group differences that may be influenced by stage and disease progression.

### Exploratory internally cross-validated modeling analysis of bacteria-only feature panels

**Figure 3:**
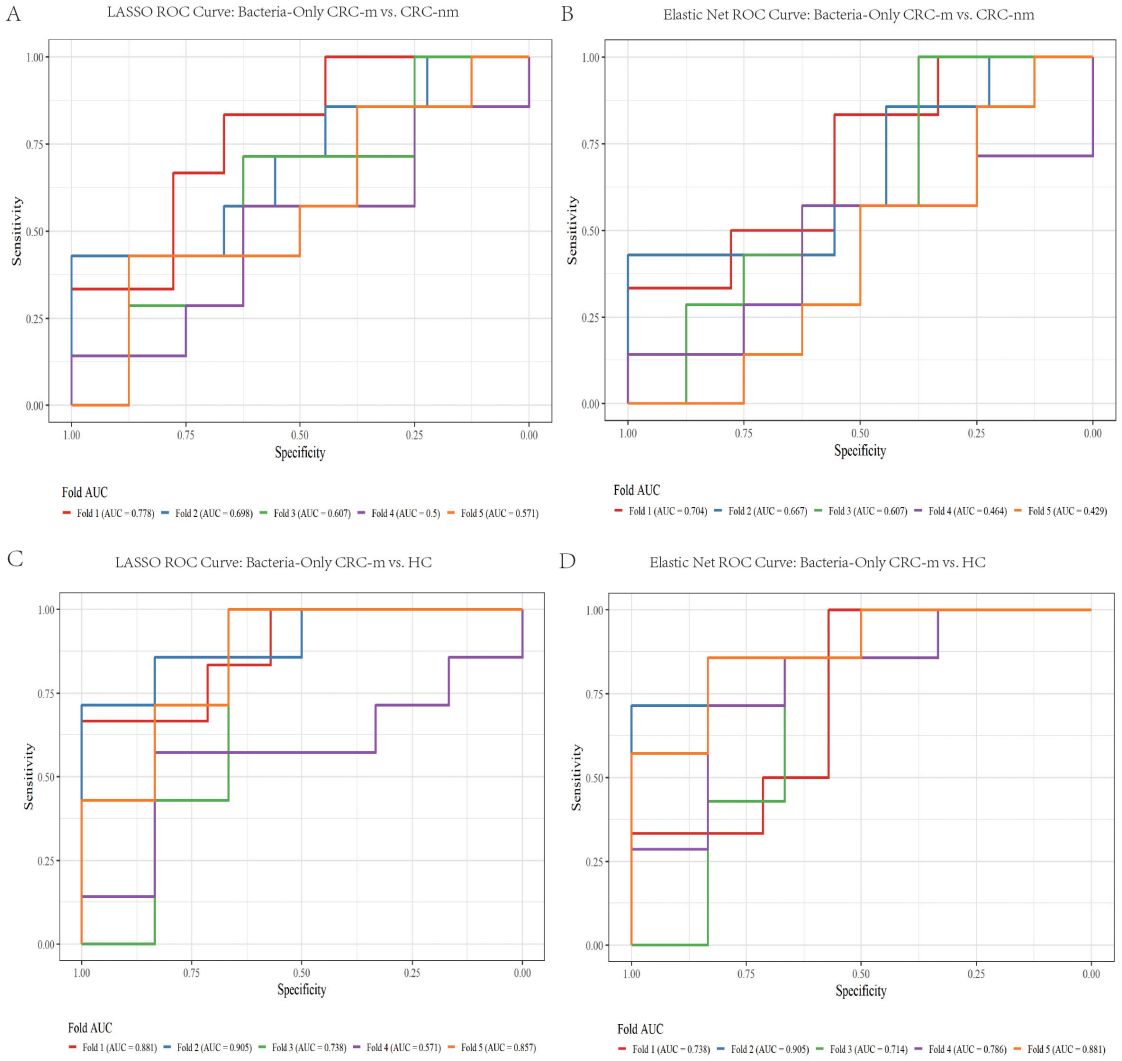
Exploratory out-of-fold ROC analysis of bacteria-only feature panels. **(A, B)** Fold-specific ROC curves for the bacteria-only CRC-m vs. CRC-nm comparison using LASSO logistic regression **(A)** and elastic net logistic regression **(B)**. **(C, D)** Fold-specific ROC curves for the bacteria-only CRC-m vs. HC comparison using LASSO logistic regression **(C)** and elastic net logistic regression **(D)**. Models were evaluated using an outer five-fold stratified cross-validation framework. Within each outer training fold, species-level bacterial abundance features were filtered using prevalence >10% and mean relative abundance >0.01%, and the resulting training-fold-specific feature set was applied to the corresponding held-out validation fold. LASSO and elastic net logistic-regression models were fitted and tuned within the outer training folds, and model performance was evaluated only in the held-out validation folds. Each colored curve represents one outer validation fold, and the corresponding fold-level ROC-AUC is shown in the legend. The red dashed diagonal indicates the reference line for random classification.

To evaluate the exploratory discriminatory ability of species-level bacterial abundance profiles, bacteria-only models were constructed using an outer five-fold stratified cross-validation framework. Within each outer training fold, bacterial features were filtered according to the predefined prevalence and mean-relative-abundance criteria, and the resulting training-fold-derived feature set was then applied unchanged to the corresponding held-out validation fold. LASSO and elastic net logistic-regression models were fitted and tuned within the training folds, whereas the held-out validation folds were used exclusively for performance evaluation. Fold-level model performance and aggregated selected-feature summaries for the bacteria-only models, including selection frequency, mean non-zero coefficient, and coefficient direction, are provided in **Supplementary Table 12**, and the corresponding ROC curves across the five validation folds are shown in **Figure 3**.

For bacteria-only models, species-level bacterial abundance features showed limited discriminative performance in distinguishing CRC-m from CRC-nm. The LASSO model achieved a mean ROC-AUC of 0.631 ± 0.109, while elastic net showed similar performance (ROC-AUC = 0.574 ± 0.122). Overall, bacterial features demonstrated weak discriminatory ability between CRC-m and CRC-nm groups.

In the CRC-m versus HC comparison, bacteria-only models demonstrated improved performance compared with the CRC-m versus CRC-nm task. The LASSO model achieved a mean ROC-AUC of 0.790 ± 0.138, and elastic net showed comparable performance. These results indicate stronger disease-associated signals than within-CRC stratification.

These findings indicate that bacterial abundance profiles capture disease-associated signals more effectively than progression-stage differences within CRC. Overall, these exploratory internal cross-validation results indicate that species-level bacterial abundance features may capture CRC-associated microbial alterations, whereas their ability to discriminate metastatic from non-metastatic CRC remains limited.

### Serum metabolite differences across clinical groups

**Figure 4:**
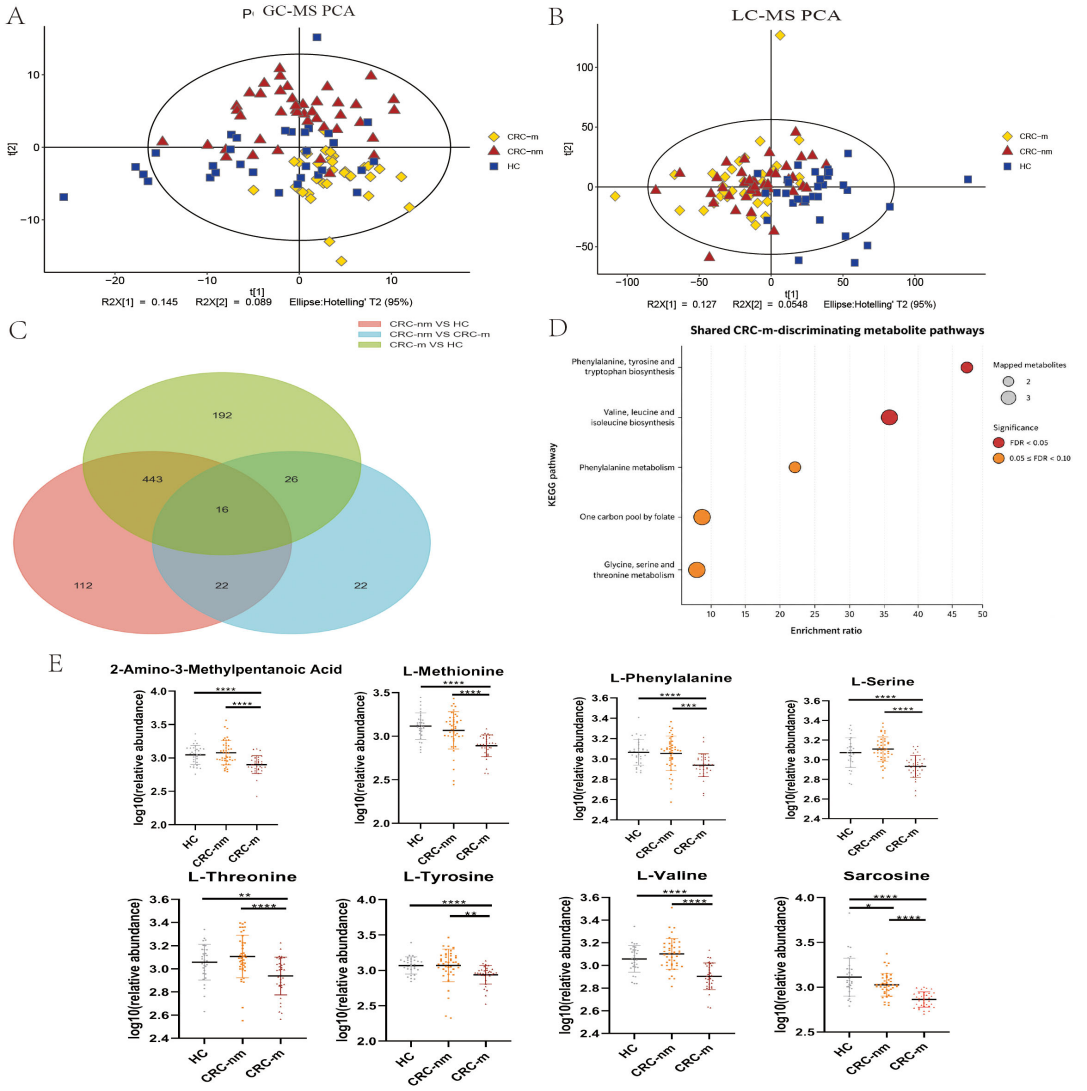
Overview of serum metabolites and KEGG pathway enrichment. **(A, B)** Unsupervised PCA score plots of GC-MS **(A)** and LC-MS **(B)** metabolomics data across HC, CRC-nm, and CRC-m groups. **(C)** Venn diagram showing the overlap of differential metabolites identified in the CRC-m vs. CRC-nm and CRC-m vs. HC comparisons. Metabolites shared by the CRC-m vs. CRC-nm and CRC-m vs. HC comparisons were defined as a conservative shared metabolite set identified in comparisons involving the CRC-m group for exploratory pathway-oriented analysis. This intersected metabolite set was not interpreted as metastasis-specific. **(D)** KEGG pathway-based enrichment analysis of the shared metabolite set identified in comparisons involving the CRC-m group. The analysis was used as an exploratory pathway-level summary rather than as evidence of metastasis-specific pathway dysregulation. The x-axis represents the enrichment ratio, bubble size indicates the number of mapped metabolites, and bubble color indicates the FDR-based significance category. Pathway significance is indicated by categorical coloring based on statistical thresholds: red indicates FDR < 0.05 (statistically significant), orange indicates 0.05 ≤ FDR < 0.10 (borderline enrichment). Continuous color gradients were not used to avoid overinterpretation of non-significant differences. **(E)** Relative abundance of the eight metabolites mapped to the significantly or borderline enriched pathways. PLS-DA score plots and overfitting assessments are provided in **Supplementary Figures 1**, **2** as supervised complementary analyses. *p < 0.05, **p < 0.01, ***p < 0.001, ****p < 0.0001.

Untargeted LC-MS and GC-MS metabolomic analyses were performed to identify serum metabolites differing across HC, CRC-nm, and CRC-m groups in the present cohort. To provide an unsupervised overview of the metabolomics data, principal component analysis (PCA) score plots for GC-MS (**Figure 4A**) and LC-MS (**Figure 4B**) were presented in the main text. The PCA plots showed partial separation among the three groups, although substantial overlap remained. Complementary supervised analyses, including PLS-DA score plots and model overfitting assessments based on 7-fold cross-validation and 200 response permutations, are shown in **Supplementary Figures 1**, **2**. In addition, pooled QC-based quality-control assessments supported acceptable overall analytical stability and reproducibility of both metabolomics platforms, including total ion chromatogram (TIC) overlap, intensity distribution, principal component analysis (PCA), and hierarchical clustering for GC-MS (**Supplementary Figure 3**), intensity distribution, PCA, and hierarchical clustering for LC-MS (**Supplementary Figure 4**), and sample-level metabolite intensity distributions before and after correction for both GC-MS and LC-MS datasets (**Supplementary Figure 5**). In **Supplementary Figure 5**, sample-level log10-transformed metabolite intensity distributions are shown before and after correction for GC-MS (**Supplementary Figures 5A, B**) and LC-MS (**Supplementary Figures 5C, D**). For the LC-MS dataset, **Supplementary Figure 5C** represents the Progenesis QI-normalized and log10-transformed matrix before QC-based LOESS signal-drift correction, statTarget batch correction, and missing-value imputation, whereas **Supplementary Figure 5D** represents the corresponding matrix after LOESS correction, statTarget batch correction, and KNN-based missing-value imputation. The dense low-intensity component observed in **Supplementary Figure 5D**, with log10-transformed values around approximately −6 to −8, mainly reflected small positive values generated during KNN-based imputation for features whose original signals were below the detection limit or absent in some samples. These values were retained in downstream differential metabolite testing after preprocessing, correction, imputation, and QC filtering. Therefore, this low-intensity component was interpreted as a preprocessing- and imputation-related feature of the corrected LC-MS matrix rather than as an independent biological signal. Study-sample injection order was randomized, QC samples were injected at regular intervals, QC-based LOESS signal-drift correction was applied to LC-MS data, LC-MS and GC-MS data matrices were separately batch-corrected using statTarget, and ion peaks with QC RSD >30% were removed before downstream analysis. Feature-level QC evidence was further available for the pathway-mapped metabolites carried forward into the main results. After correction, the QC RSD values of the eight reported metabolites ranged from 1.45% to 3.64%, and the internal-standard QC RSD values were 6.28% in negative-ion mode and 2.10% in positive-ion mode, supporting acceptable analytical reproducibility of the reported metabolite features and internal-standard signals (**Supplementary Table 9**). Nevertheless, because blank raw data were not retained and the LC-MS normalization target was limited to the Progenesis QI default workflow description, the metabolomics results were still interpreted cautiously as cross-sectional patterns under the present preprocessing workflow.

These results suggest cross-sectional serum metabolite differences among the clinically defined groups under the current analytical workflow, with partial group separation but without establishing metabolite alterations attributable specifically to metastatic dissemination.

To identify metabolites that consistently differed in the CRC-m group relative to both comparison groups, pairwise comparisons were performed between CRC-m and CRC-nm and between CRC-m and HC using LC-MS and GC-MS data, with differential metabolites defined by VIP > 1, Benjamini–Hochberg FDR-adjusted p < 0.05, and fold change > 1.2 or < 0.83; complete comparison-specific statistical outputs are provided in **Supplementary Tables 5, 6**. The overlap between these two pairwise comparisons was defined as a conservative shared metabolite set identified in comparisons involving the CRC-m group for pathway-oriented exploratory analysis (**Figure 4C**). This intersected set was used to summarize metabolic patterns consistently observed in the CRC-m group relative to CRC-nm and HC, rather than to define metastasis-specific metabolites. Because CRC-m and CRC-nm also differed by stage, the shared metabolite set should be interpreted as reflecting stage- and progression-confounded cross-sectional group differences.

To provide a pathway-level summary of the shared metabolite set identified in comparisons involving the CRC-m group, these metabolites were subjected to KEGG pathway-based enrichment analysis. This analysis was intended as an exploratory pathway-oriented summary of the selected metabolite set rather than as a test of metastasis-specific pathway dysregulation. Rather than selecting pathways based on an arbitrary ranking cutoff, all enriched pathways were evaluated according to predefined statistical criteria. Two pathways remained significant after FDR correction (FDR < 0.05), namely valine, leucine and isoleucine biosynthesis and phenylalanine, tyrosine and tryptophan biosynthesis, whereas three additional pathways showed borderline enrichment (0.05 ≤ FDR < 0.10), including one carbon pool by folate, phenylalanine metabolism, and glycine, serine and threonine metabolism (**Supplementary Table 7**; **Figure 4D**).

These five pathways were therefore retained for downstream interpretation because they collectively represent all pathways with statistically supported enrichment signals under the defined thresholds, rather than being selected based on arbitrary ranking. In addition, these pathways converge on a coherent biological theme centered on branched-chain amino acid metabolism, aromatic amino acid metabolism, and one-carbon-related metabolism.

Within these five statistically supported or borderline enriched pathways, eight metabolites were mapped, including L-phenylalanine, L-tyrosine, L-valine, 2-amino-3-methylpentanoic acid, sarcosine, L-methionine, L-serine, and L-threonine (**Supplementary Table 8**). As shown in **Figure 4E**, these eight pathway-mapped metabolites showed lower measured abundance in the CRC-m group than in both the CRC-nm and HC groups after preprocessing and statistical filtering, and each showed significant pairwise differences between CRC-m and CRC-nm and between CRC-m and HC. However, because the metabolite set was selected from cross-sectional pairwise comparisons, LC-MS normalization was limited to the Progenesis QI default workflow description, blank raw data were not retained, and metabolite identities were putatively annotated rather than confirmed by authentic standards, these findings should be interpreted as a descriptive lower-abundance pattern of selected metabolites under the present workflow rather than as evidence of selective biological depletion or metastasis-specific metabolic alteration.

Collectively, these findings indicate a cross-sectional lower-abundance pattern of a selected metabolite subset in the CRC-m group under the present workflow, rather than evidence that these metabolic differences are attributable specifically to metastatic dissemination.

### Exploratory internally cross-validated modeling analysis of serum metabolite feature panels

**Figure 5:**
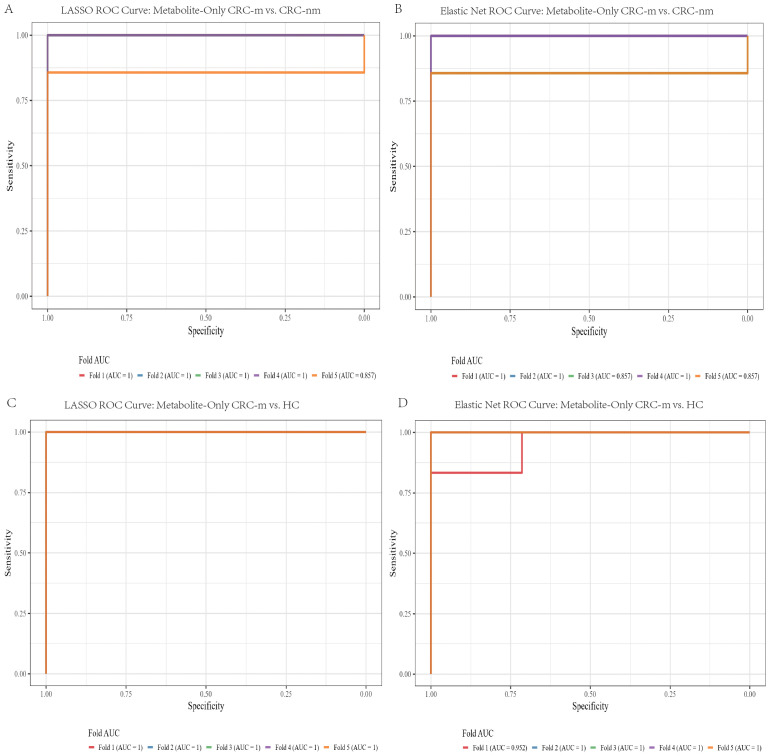
Exploratory out-of-fold ROC analysis of metabolite-only feature panels. **(A, B)** Fold-specific ROC curves for the metabolite-only CRC-m vs. CRC-nm comparison using LASSO logistic regression **(A)** and elastic net logistic regression **(B)**. **(C, D)** Fold-specific ROC curves for the metabolite-only CRC-m vs. HC comparison using LASSO logistic regression **(C)** and elastic net logistic regression **(D)**. Models were evaluated using an outer five-fold stratified cross-validation framework. All detected serum metabolite features were used without additional univariate or differential-metabolite prescreening before penalized regression. LASSO and elastic net logistic-regression models were fitted and tuned within the outer training folds, and model performance was evaluated only in the held-out validation folds. Each colored curve represents one outer validation fold, and the corresponding fold-level ROC-AUC is shown in the legend. The red dashed diagonal indicates the reference line for random classification.

Exploratory internally cross-validated modeling analysis of serum metabolite feature panels was conducted using the same outer five-fold stratified cross-validation framework. All detected serum metabolite features were included in the penalized regression models without additional univariate or differential-metabolite prescreening. Within each outer training fold, model fitting, tuning, and feature selection were performed exclusively on the training set, while the held-out validation fold was used solely for performance evaluation. Fold-level model performance and aggregated selected-feature summaries for the metabolite-only models, including selection frequency, mean non-zero coefficient, and coefficient direction, are provided in **Supplementary Table 13**, and the corresponding ROC curves across the five validation folds are shown in **Figure 5**.

For metabolite-only models, both LASSO and elastic net models achieved strong discriminatory performance in distinguishing CRC-m from CRC-nm. The LASSO model reached a mean ROC-AUC of 0.971 ± 0.064, while elastic net showed slightly reduced but robust performance. Overall, serum metabolite profiles provided strong and stable discrimination between CRC-m and CRC-nm groups.

In the CRC-m versus HC comparison, metabolite-only models achieved near-perfect classification performance. Both LASSO and elastic net models showed strong discriminative ability, indicating robust metabolic separation between groups.

### *Post hoc* selected-feature exploratory within-group correlations between LEfSe-selected candidate microbial species and pathway-mapped metabolites

**Figure 6:**
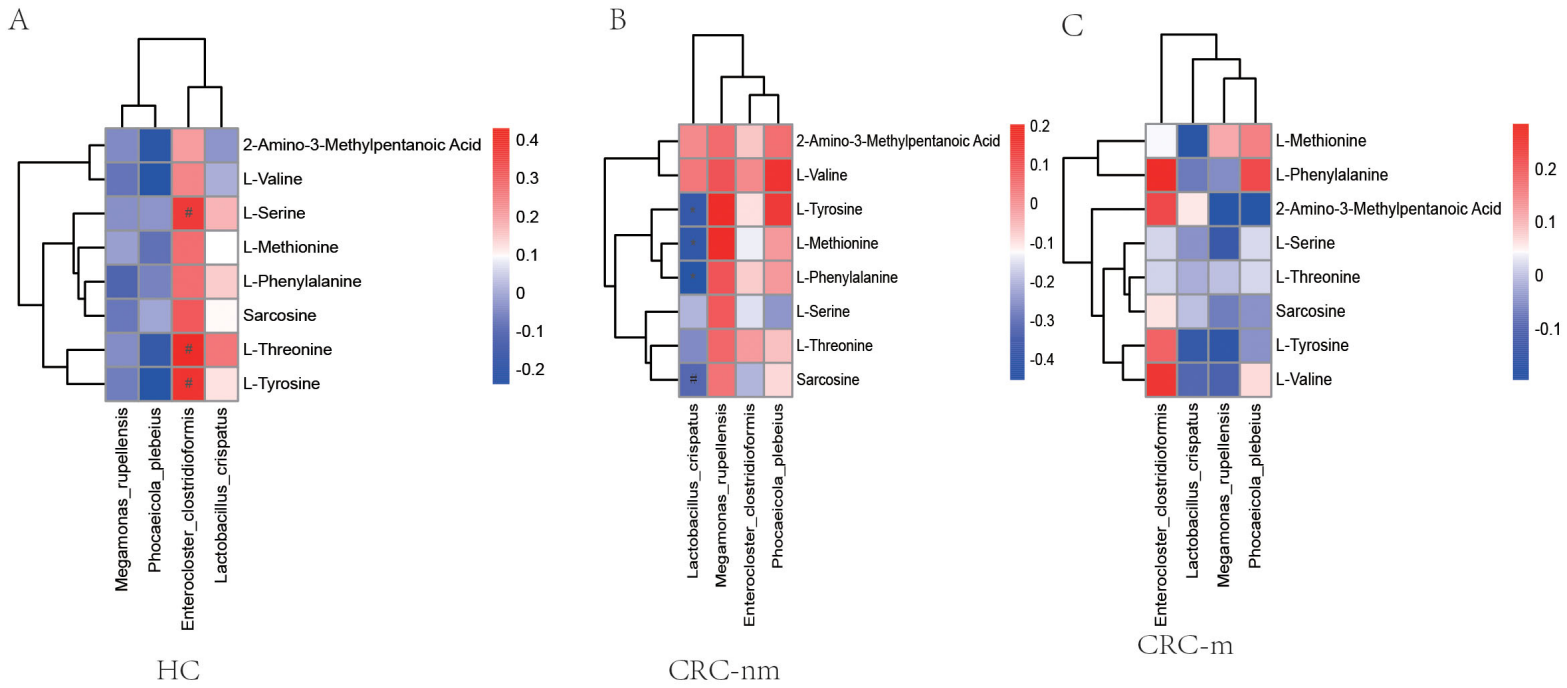
*Post hoc* selected-feature exploratory within-group correlations between LEfSe-selected candidate microbial species and pathway-mapped metabolites. *Post hoc* selected-feature exploratory Spearman correlation analyses were performed separately in the HC, CRC-nm, and CRC-m groups between the four retained candidate species-level features and the eight pathway-mapped metabolites. Heatmap panels **(A–C)** correspond to HC **(A)**, CRC-nm **(B)**, and CRC-m **(C)**, respectively. Heatmap colors indicate Spearman’s correlation coefficients. P values were adjusted using the Benjamini–Hochberg false discovery rate procedure within each group-specific correlation set. Significance symbols indicate adjusted p values: *adjusted p <0.05 and #0.05 ≤ adjusted p <0.10. No symbol indicates adjusted p ≥0.10.

To further explore potential microbiota–metabolite relationships within a limited preselected feature set, a *post hoc* selected-feature exploratory correlation analysis was performed within each clinical group between the four retained LEfSe-selected candidate species-level features (Enterocloster clostridioformis, Lactobacillus crispatus, Megamonas rupellensis, and Phocaeicola plebeius) and the eight selected pathway-mapped metabolites (**Supplementary Table 10**). Additionally, similar *post hoc* selected-feature exploratory correlation analyses were conducted between these four candidate species and the systemic inflammatory indices (**Supplementary Table 11**). The analyses were performed separately in the HC, CRC-nm, and CRC-m groups, and p values were adjusted using the Benjamini–Hochberg false discovery rate procedure within each group-specific correlation set (**Figure 6**).

In the HC group, Enterocloster clostridioformis showed borderline positive correlations with L-serine, L-tyrosine, and L-threonine after adjustment, whereas no adjusted significant associations were observed. In the CRC-nm group, Lactobacillus crispatus showed negative correlations with L-phenylalanine, L-methionine, and L-tyrosine after adjustment. A borderline negative correlation between Lactobacillus crispatus and sarcosine was also observed. In contrast, no adjusted significant or borderline associations were detected in the CRC-m group.

Overall, this limited *post hoc* selected-feature correlation analysis identified only limited and group-specific taxa–metabolite associations. These findings were interpreted as exploratory because of the small within-group sample sizes, upstream feature preselection, limited correlation sets, and the cross-sectional study design. Therefore, the correlation results were considered supportive exploratory observations rather than evidence of mechanistic microbiota–metabolite interactions.

### Exploratory internally cross-validated modeling analysis of integrated microbiota–metabolite feature panels

**Figure 7:**
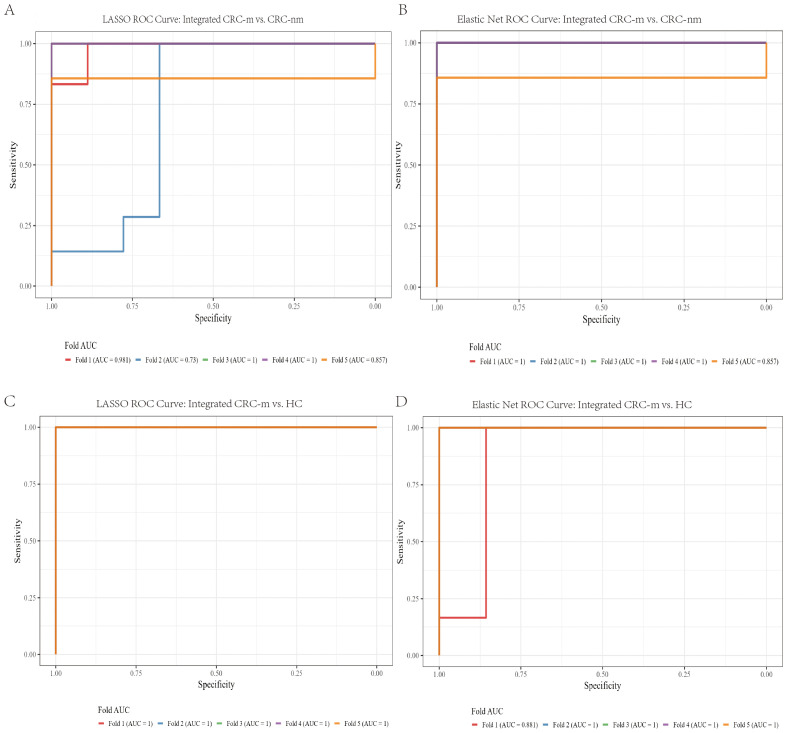
Exploratory out-of-fold ROC analysis of integrated microbiota–metabolite feature panels. **(A, B)** Fold-specific ROC curves for the integrated CRC-m vs. CRC-nm comparison using LASSO logistic regression **(A)** and elastic net logistic regression **(B)**. **(C, D)** Fold-specific ROC curves for the integrated CRC-m vs. HC comparison using LASSO logistic regression **(C)** and elastic net logistic regression **(D)**. Models were evaluated using an outer five-fold stratified cross-validation framework. Within each outer training fold, bacterial features were filtered using prevalence >10% and mean relative abundance >0.01%, and the resulting training-fold-specific feature set was applied to the corresponding held-out validation fold. All detected serum metabolite features were retained without additional univariate or differential-metabolite prescreening. The training-fold-filtered bacterial matrix and metabolite matrix were combined within each outer fold before penalized logistic-regression modeling. LASSO and elastic net logistic-regression models were fitted and tuned within the outer training folds, and model performance was evaluated only in the held-out validation folds. Each colored curve represents one outer validation fold, and the corresponding fold-level ROC-AUC is shown in the legend. The red dashed diagonal indicates the reference line for random classification.

To evaluate exploratory performance after integrating microbial and metabolic features, combined microbiota–metabolite models were developed using the same outer five-fold stratified cross-validation framework. In each outer training fold, bacterial features were filtered based on predefined prevalence and mean-relative-abundance criteria, and the resulting bacterial feature set was applied to the corresponding validation fold. Serum metabolite features were retained without additional prescreening. The training-fold-filtered bacterial matrix and metabolite matrix were then concatenated prior to penalized logistic-regression modeling. LASSO and elastic net models were fitted, tuned, and used for feature selection within training folds, whereas held-out validation folds were used only for performance evaluation. For elastic net, candidate alpha values from 0.1 to 0.9 were evaluated, and for each alpha value, lambda was selected using five-fold internal cross-validation with ROC-AUC as the tuning criterion. Fold-level model performance and aggregated selected-feature summaries for the integrated microbiota–metabolite models, including selection frequency, mean non-zero coefficient, and coefficient direction, are provided in **Supplementary Table 14**, and the corresponding ROC curves across the five validation folds are shown in **Figure 7**.

For integrated microbiota–metabolite models, internal discrimination remained high in distinguishing CRC-m from CRC-nm; however, the addition of bacterial features did not improve performance over the corresponding metabolite-only models. In the clinically most relevant CRC-m versus CRC-nm comparison, the integrated LASSO model achieved a mean ROC-AUC of 0.914 ± 0.119, which was lower than that of the metabolite-only LASSO model. In addition, the integrated LASSO model showed lower PR-AUC, sensitivity, NPV, and F1-score than the corresponding metabolite-only LASSO model. The integrated elastic net model reached a high ROC-AUC, but several threshold-dependent metrics were lower than those of the corresponding metabolite-only elastic net model despite higher ROC-AUC/PR-AUC. Therefore, the integrated models should not be interpreted as equivalent or preferable to the metabolite-only models in this comparison.

In the CRC-m versus HC comparison, integrated models yielded high absolute internal performance estimates, but they did not demonstrate a clear or consistent improvement over the corresponding metabolite-only models. Across the evaluated pairwise comparisons, adding bacterial features provided no incremental predictive benefit over metabolite-only models and, for some model/comparison/metric combinations, was associated with lower internal performance. These results suggest that the discriminatory information was mainly captured by serum metabolite features, whereas bacterial features did not add measurable predictive value in the present exploratory internal validation workflow.

### Groupwise differences in inflammatory response indices across HC, CRC-nm, and CRC-m groups

**Figure 8:**
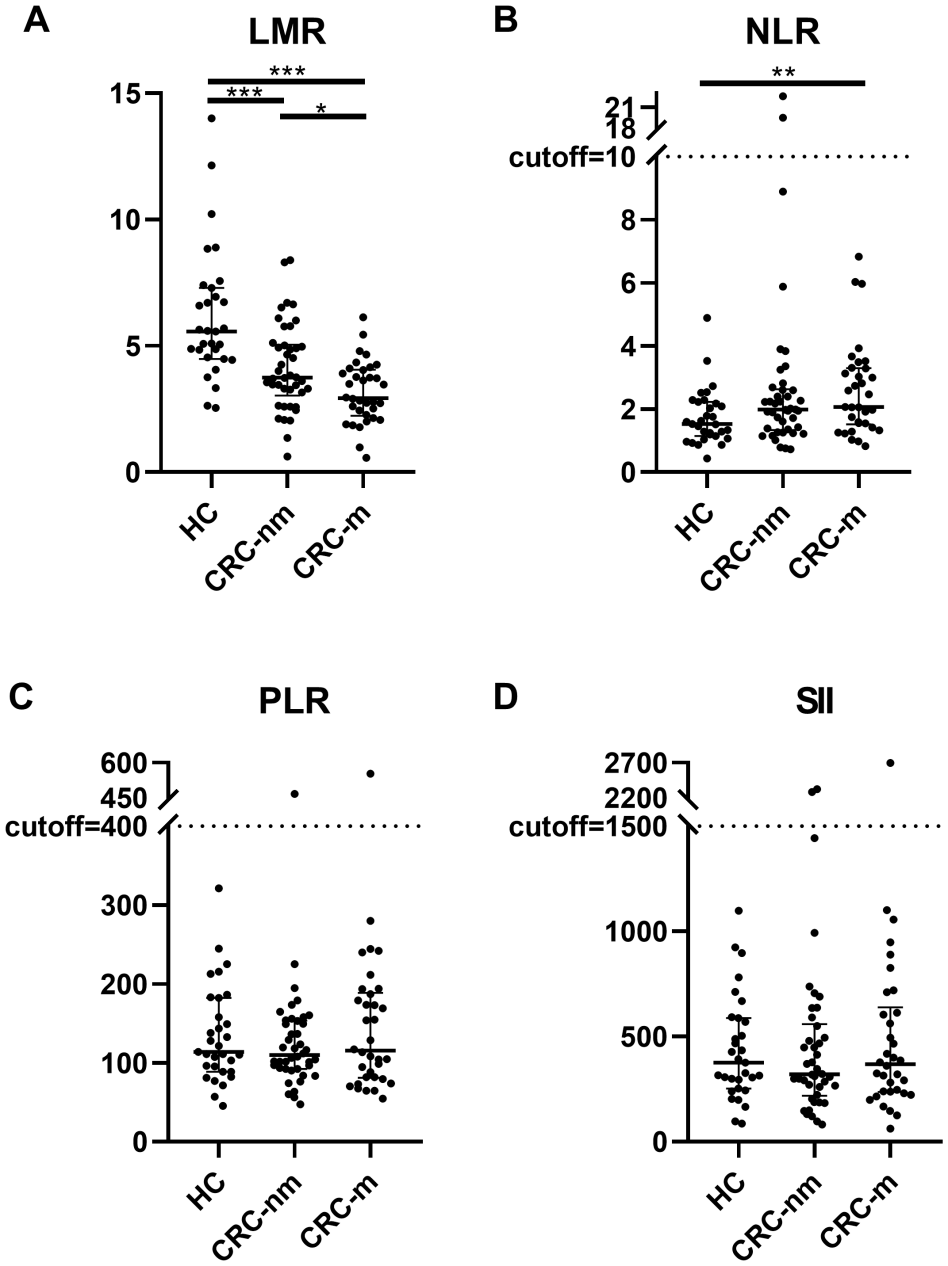
Inflammatory response indices in HC, CRC-nm, and CRC-m groups. Inflammatory response indices (NLR, LMR, PLR, and SII) in HC, CRC-nm, and CRC-m groups. Panels **(A–D)** correspond to LMR **(A)**, NLR **(B)**, PLR **(C)**, and SII **(D)**, respectively. Exploratory within-group Spearman correlations between selected microbial/metabolite features and inflammatory indices are provided in **Supplementary Table 11**.

Inflammation is closely linked to tumor development. To descriptively compare systemic inflammatory status across the three clinically defined groups, we collected neutrophil, lymphocyte, monocyte, and platelet counts for each participant. Neutrophil-to-lymphocyte ratio (NLR), lymphocyte-to-monocyte ratio (LMR), platelet-to-lymphocyte ratio (PLR), and systemic immune-inflammation index (SII) were calculated (**Supplementary Table 1**). LMR showed a decreasing pattern across HC, CRC-nm, and CRC-m groups and differed significantly among groups, whereas NLR showed an increasing pattern; significant differences in NLR were observed only between the HC and CRC-nm groups. PLR and SII did not differ significantly among the three groups (**Figure 8**).

## Discussion

This study characterized cross-sectional differences in gut microbiota, serum metabolites, and pathway-oriented metabolic signals across HC, CRC-nm, and CRC-m groups. Given that CRC-m and CRC-nm differed by disease stage, observed differences should be interpreted primarily as stage- and disease-burden-associated variations rather than metastasis-specific biological signatures. Group-level differences were observed in microbial features, metabolites, and pathways across the three clinically defined groups. However, these differences should be interpreted in the context of a stage-confounded cross-sectional design rather than as disease-specific mechanistic alterations. Because all CRC-m cases were stage IV and all CRC-nm cases were stages I–III, these differences may reflect disease stage, tumor burden, current disease status, or broader progression-related changes.

We evaluated bacteria-only, metabolite-only, and integrated microbiota–metabolite feature panels using an outer five-fold stratified cross-validation framework. These analyses were exploratory internal validation procedures and were not intended to establish clinically generalizable predictive models. For bacteria-only and integrated models, species-level bacterial abundance features were filtered within each outer training fold. However, given the cross-validation-only design and lack of external validation, results should be interpreted as exploratory pattern recognition rather than robust predictive modeling. For metabolite-only models, detected serum metabolite features were used without additional univariate or differential-metabolite prescreening. For integrated models, the training-fold-filtered bacterial matrix and metabolite matrix were combined before penalized logistic-regression modeling. Model tuning, fitting, and model-based feature selection were performed only within the outer training folds, whereas the held-out validation folds were used exclusively for performance evaluation. ROC-AUC, PR-AUC, sensitivity, specificity, PPV, NPV, and F1-score were reported as exploratory internal cross-validation estimates. These analyses should be interpreted as exploratory internal validation rather than evidence of clinically established diagnostic performance, because no independent external validation cohort was available and CRC-m and CRC-nm were completely confounded by disease stage.

An increasing number of studies have examined gut microbial profiles as candidate features for colorectal cancer detection, stratification, and biological interpretation. Large-scale metagenomic analyses have shown that CRC-associated microbial signatures can vary according to disease stage, tumor location, cohort background, and taxonomic or strain-level resolution, supporting the need for cautious interpretation of species-level findings in individual cohorts (Chen et al., 2022; Fidelle et al., 2023; Zepeda-Rivera et al., 2024; Piccinno et al., 2025). In the present study, species-level microbial features were analyzed after retaining unambiguously annotated species with a mean relative abundance greater than 0.0001 and a prevalence greater than 10%. LEfSe analysis was then performed using the prespecified exploratory criteria of an FDR-adjusted p value <0.10 and an LDA score >2.5. Under these exploratory criteria, *Enterocloster clostridioformis* and *Lactobacillus crispatus* were retained as shared CRC-m-enriched candidate species-level features, while *Megamonas rupellensis* and *Phocaeicola plebeius* were identified as shared CRC-m-depleted features across both comparisons. Crucially, in the clinically most relevant CRC-m vs. CRC-nm contrast, the two enriched species passed only the relaxed exploratory FDR < 0.10 threshold, failing to meet the conventional FDR < 0.05 level. Therefore, these taxa should not be described as statistically significant or validated CRC-m-associated microbial markers, but interpreted strictly as exploratory candidate features identified under a relaxed FDR threshold.

The retention of Enterocloster clostridioformis as an exploratory candidate feature is noteworthy, but should be interpreted cautiously, because recent mechanistic work has suggested that Enterocloster species can participate in gut microbiota–immune interactions in cancer-related contexts. Fidelle et al. reported that post-antibiotic recolonization by Enterocloster species could downregulate ileal MAdCAM-1, promote the migration of gut-derived immunosuppressive T cells into tumors, and compromise antitumor immune responses in the setting of immune checkpoint blockade (Fidelle et al., 2023). However, that study was not designed to establish a role for Enterocloster clostridioformis in CRC metastasis, and its findings should be used only as biological context. In the present cohort, the higher relative abundance of Enterocloster clostridioformis in the CRC-m/stage IV group should therefore be interpreted as a cross-sectional microbial signal associated with advanced clinical status rather than as evidence that this species directly promotes metastatic dissemination.

Lactobacillus crispatus was also retained as a CRC-m-enriched candidate species-level feature shared by both pairwise comparisons. Members of the Lactobacillus genus are often discussed in relation to mucosal homeostasis, probiotic activity, inflammation regulation, and cancer-related host–microbe interactions, but their effects are highly species-, strain-, niche-, and disease-context dependent (Decout et al., 2024; Li et al., 2024). Therefore, enrichment of Lactobacillus crispatus in the CRC-m/stage IV group should not be interpreted as a simple protective or pathogenic effect. Instead, it may reflect altered gut ecological conditions in advanced CRC, differences in host physiology, tumor burden, diet-related exposures, inflammation, or other unmeasured clinical factors. Because direct evidence linking Lactobacillus crispatus to CRC metastatic biology remains limited, this result should be considered an exploratory candidate association requiring independent validation.

The analysis of shared altered features across both enrichment and depletion directions provided a broader view of microbial differences across the clinical groups. In addition to CRC-m-enriched taxa, consistently altered taxa in the opposite direction were reported to provide complementary biological context. Recent experimental studies have highlighted the potentially protective role of Phocaeicola plebeius (formerly Bacteroides plebeius); for instance, its gut colonization has been shown to restructure the microbial community, produce beneficial metabolites, and actively suppress the development of colitis-associated colon cancer in murine models (Chen et al., 2025). Thus, its lower abundance in the CRC-m/stage IV group may be consistent with reduced representation of potentially protective microbial functions during advanced disease. Similarly, Megamonas species, including Megamonas rupellensis, are recognized as important commensals involved in host metabolic regulation and the generation of short-chain fatty acids (SCFAs), which are vital for maintaining intestinal barrier integrity and modulating inflammation (Chen et al., 2022). Megamonas rupellensis showed a relatively stronger shared alteration, meeting the conventional FDR < 0.05 threshold across both CRC-m comparisons. Selected-feature exploratory correlation analyses did not identify consistent associations involving the CRC-m-depleted candidate species, and their functional implications therefore remain uncertain in this cohort. These depletion patterns suggest that the gut ecosystem in advanced CRC may involve both expansion of potential pathobionts and reduced abundance of commensals associated with immunological and metabolic homeostasis. Together, the enrichment and depletion feature panels (**Figure 2**) provide a more balanced view of gut ecological shifts across the clinically defined groups.

Nevertheless, these microbial findings should be interpreted within the main design limitation of the study. All CRC-m cases were stage IV, whereas all CRC-nm cases were stages I–III. Therefore, the observed microbial differences cannot be separated from disease-stage confounding. The enrichment of Enterocloster clostridioformis and Lactobacillus crispatus may reflect stage-related, tumor burden-related, or broader progression-associated ecological changes rather than microbiota changes specifically attributable to metastatic dissemination. This distinction is important because recent large-scale studies emphasize that CRC-associated microbiome signatures may show stage-dependent and strain-level patterns, and that species-level associations from individual cohorts require validation before being considered clinically or mechanistically robust (Fidelle et al., 2023; Piccinno et al., 2025).

Overall, the exploratory LEfSe analysis identified Enterocloster clostridioformis and Lactobacillus crispatus as shared CRC-m-enriched candidate species-level features, and Megamonas rupellensis and Phocaeicola plebeius as shared CRC-m-depleted candidate species-level features under the prespecified FDR-adjusted p <0.10 and LDA criteria. These findings describe candidate microbial differences across clinically defined CRC groups, but further validation is required to clarify their biological and clinical significance. Larger, independent, stage-balanced, and preferably longitudinal cohorts, together with strain-level metagenomic analysis and functional experiments, will be needed to determine whether these microbial features are reproducibly associated with advanced CRC status and whether they have any direct biological relevance to CRC progression.

Pathway-based metabolite set enrichment analysis was used here as an exploratory pathway-oriented summary of the shared metabolite set identified in comparisons involving the CRC-m/stage IV group. This metabolite set was defined by the intersection of CRC-m vs. CRC-nm and CRC-m vs. HC comparisons to summarize metabolites that differed in the CRC-m/stage IV group relative to both comparator groups. However, this strategy does not identify metastasis-specific metabolites, because the CRC-m vs. CRC-nm comparison was confounded by disease stage and the CRC-m vs. HC comparison captured broader disease-vs-healthy differences. Because the metabolite set entering the enrichment analysis was largely composed of amino acids and related compounds, enrichment of amino acid-related pathways was not unexpected and should not by itself be interpreted as a strong mechanistic finding. Instead, the enrichment results should be regarded as a descriptive summary indicating that the selected stage-/status-associated cross-sectional metabolite differences were concentrated in branched-chain amino acid-related and one-carbon-related pathways (Yang and Vousden, 2016; Chen et al., 2024; Lee et al., 2024).

Among the mapped metabolites, the lower measured abundance of L-valine and 2-amino-3-methylpentanoic acid placed the selected metabolite pattern within branched-chain amino acid-related metabolism, which has been discussed in cancer biology and advanced CRC-related contexts (Tian et al., 2020; Chen et al., 2024). Likewise, the lower measured abundance of L-serine, L-methionine, L-threonine, and sarcosine placed the selected metabolite pattern within one-carbon-associated metabolism, which is widely involved in nucleotide synthesis, methylation, and redox-related processes in cancer (Yang and Vousden, 2016; Lee et al., 2024). Taken together, these findings suggest a cross-sectional metabolite pattern related to amino acid and one-carbon-associated metabolism in the CRC-m/stage IV group. However, because the present analysis is based on cross-sectional abundance differences and pathway over-representation rather than functional assays, flux measurements, or longitudinal sampling, these results should be interpreted as descriptive pathway-oriented evidence rather than direct evidence of mechanistic pathway dysregulation driving CRC progression or metastatic dissemination. More specifically, the present findings should be distinguished from metastasis-specific evidence: they describe cross-sectional metabolic differences in the CRC-m/stage IV group within this cohort, set against a broader background of cancer-related amino acid and one-carbon metabolism, but do not establish metabolic mechanisms unique to metastatic dissemination. Importantly, the reported compounds were putatively annotated rather than fully confirmed by authentic standards. Therefore, the present pathway-level interpretation should be regarded as exploratory and descriptive rather than as definitive metabolite-level identification or mechanism-level evidence.

Importantly, pooled QC-based assessments supported acceptable overall analytical stability of the metabolomics workflow, with GC-MS evaluated by TIC overlap, intensity distribution, PCA, and hierarchical clustering (**Supplementary Figure 3**), and LC-MS evaluated by intensity distribution, PCA, and hierarchical clustering (**Supplementary Figure 4**). Study-sample injection order was randomized, pooled QC samples were injected at regular intervals, QC-based LOESS signal-drift correction was applied to LC-MS data, LC-MS and GC-MS data matrices were separately batch-corrected using statTarget, and features with QC RSD >30% were removed before downstream analysis.

Sample-level intensity distribution plots before and after correction were also provided for both GC-MS and LC-MS datasets (**Supplementary Figure 5**), offering additional visualization of the preprocessing and correction workflow. Additional feature-level QC evidence was available for the metabolites carried forward into the pathway-oriented analysis. After correction, the QC RSD values of the eight pathway-mapped metabolites ranged from 1.45% to 3.64%, and the internal-standard QC RSD values were 6.28% in negative-ion mode and 2.10% in positive-ion mode. These data provide more direct support for analytical reproducibility of the reported metabolite features than pooled-QC plots alone. Nevertheless, several technical limitations remain, including the use of Progenesis QI default LC-MS normalization without a fully specified normalization target at the manuscript level, the lack of retained blank raw data for documenting background-peak or contaminant removal, and putative rather than authentic-standard-confirmed metabolite annotation. Therefore, the pathway-level interpretation remains exploratory and descriptive rather than definitive mechanistic evidence.

The within-group correlation analyses provided only limited exploratory evidence for microbiota–metabolite associations. In the CRC-nm group, Lactobacillus crispatus showed negative correlations with several amino acid-related metabolites, including L-phenylalanine, L-methionine, and L-tyrosine, whereas its correlation with sarcosine showed borderline significance after adjustment. In the HC group, Enterocloster clostridioformis showed only borderline positive correlations with L-serine, L-tyrosine, and L-threonine. No adjusted significant or borderline taxa–metabolite associations were observed in the CRC-m/stage IV group. These findings suggest that the observed microbial and metabolic differences do not translate into a robust within-group correlation structure in the present cohort. Therefore, the correlation results should be interpreted as exploratory and descriptive rather than as evidence of direct microbiota–metabolite coupling or mechanistic interaction.

Given growing interest in gut microbiota and serum metabolites as candidate discriminatory features in CRC (Coker et al., 2022; Kong et al., 2023), the exploratory modeling workflow used an outer five-fold stratified cross-validation framework. In this workflow, the bacterial components of bacteria-only and integrated models were filtered within each outer training fold using prevalence >10% and mean relative abundance >0.01%, and the resulting training-fold-specific feature set was then applied to the corresponding held-out validation fold. By contrast, metabolite-only models and the metabolite component of integrated models used all detected serum metabolite features without additional univariate or differential-metabolite prescreening. After the corresponding feature matrices were defined within each fold, penalized logistic-regression models were fitted and tuned in the training folds, and their performance was evaluated only in the held-out validation folds.

Under this modeling framework, bacteria-only models showed limited internal cross-validation performance for CRC-m vs. CRC-nm, with mean ROC-AUC values of 0.631 ± 0.109 for LASSO and 0.574 ± 0.122 for elastic net. For CRC-m vs. HC, bacteria-only models yielded higher mean ROC-AUC estimates of 0.790 ± 0.138 for LASSO and 0.805 ± 0.085 for elastic net, indicating stronger discrimination between CRC-m and healthy controls than between CRC-m and CRC-nm. However, these results should still be interpreted cautiously because they were derived from internal cross-validation only and were not externally validated.

Metabolite-only models showed high internal cross-validation performance in both pairwise comparisons. By contrast, adding bacterial features did not improve predictive performance over the corresponding metabolite-only models. In the clinically most relevant CRC-m versus CRC-nm comparison, the integrated LASSO model showed lower ROC-AUC, PR-AUC, sensitivity, NPV, and F1-score than the metabolite-only LASSO model. The integrated elastic net model showed higher ROC-AUC/PR-AUC but lower threshold-dependent performance for several metrics, indicating that improved ranking-based metrics did not translate into better fixed-threshold classification performance. In the CRC-m versus HC comparison, integrated models also failed to show clear or consistent improvement over metabolite-only models. Overall, bacterial features did not provide incremental predictive value in the integrated models, and the observed discriminatory performance appeared primarily driven by the metabolite component.

These modeling results should not be interpreted as demonstrating equivalence or superiority of integrated microbiota–metabolite panels over metabolite-only panels. Rather, they indicate that integrated modeling did not yield added predictive benefit and, in some model/comparison/metric combinations, resulted in lower internal performance. This finding suggests that, under the present sample size, feature dimensionality, and cross-validation framework, adding bacterial abundance features may have introduced noise or instability rather than improving prediction. Because these results were obtained from a small, internally cross-validated cohort without external validation, they should be interpreted as descriptive internal observations rather than evidence of clinical diagnostic utility. In particular, these models discriminate clinically defined, stage-confounded groups within the present cohort; they do not establish clinically usable biomarkers or metastasis-specific discriminatory biology.

Previous studies have shown that inflammation-based systemic indices are associated with prognosis in both breast cancer and CRC, although the specific indices examined differ across studies (Chen et al., 2017; Shibutani et al., 2017; Zhu et al., 2017; Nakamoto et al., 2021). In breast cancer, low NLR, high LMR, and an elevated PLR have been linked to clinical outcomes, with an elevated PLR having been reported as a poor prognostic indicator (Zhu et al., 2017; Nakamoto et al., 2021), whereas in CRC, LMR, SII, and related inflammation-based indices have also shown prognostic relevance (Chen et al., 2017; Shibutani et al., 2017). These reports provide only broader prognostic context for the inflammatory indices examined here; they do not directly validate the cross-sectional groupwise differences observed in the present cohort.

Likewise, the current data do not support metabolite-specific functional conclusions for L-valine, sarcosine, L-methionine, L-tyrosine, or related compounds, nor do they establish a mechanistic link among gut microbiota differences, serum metabolite differences, and systemic inflammatory status. Instead, these findings should be interpreted more cautiously as stage-/status-associated cross-sectional differences in the current dataset. Further validation in larger, stage-balanced, longitudinal, and mechanistically designed studies will be needed to clarify the stability and interpretability of the microbiota-, metabolite-, and inflammation-related patterns observed in the CRC-m/stage IV group.

This study was conducted in Han Chinese patients with CRC from eastern China, and several sources of heterogeneity and residual confounding should be considered when interpreting the findings. A major limitation of this study is that all CRC-m patients were stage IV, whereas CRC-nm patients were stages I–III. Therefore, the observed differences may reflect disease stage, tumor burden, or broader progression-related changes rather than biology attributable specifically to metastatic dissemination. In addition, the main microbiota and metabolite comparisons were unadjusted group-level comparisons. Potentially relevant demographic, clinical, tumor-related, and dietary factors, including age, sex, BMI, primary tumor location, CEA level, and habitual diet, were not adjusted for in the primary group comparisons. Because some of these variables showed clinical differences or borderline differences between groups, residual confounding cannot be excluded. The cross-sectional design further supports only group-level associations at the time of sampling and does not permit inference regarding temporal sequence, future disease progression, metastatic potential, or causality. In addition, although a significant weighted UniFrac PERMANOVA group effect was observed, the complementary Bray-Curtis species-level PERMDISP analysis indicated significant heterogeneity of within-group dispersion. Therefore, the beta-diversity result should be interpreted as an exploratory global difference rather than as definitive evidence of true between-group compositional separation, because the observed PERMANOVA effect may partly reflect unequal within-group dispersion. In addition, although the LEfSe analysis was performed using predefined abundance, prevalence, annotation, FDR, and LDA thresholds, the resulting candidate species-level features should still be interpreted cautiously because the study was cross-sectional, the sample size was limited, and CRC-m and CRC-nm were completely confounded by disease stage.

For the metabolomics workflow, several limitations should still be considered despite the additional workflow clarification and feature-level QC evidence. Some platform-level statistical parameters relevant to the differential metabolite analysis, including additional scaling settings, formal normality-testing details, exact variance assumptions used in the platform t-test workflow, and other workflow-level settings, were not fully retained in the exported records, which limits complete transparency and reproducibility of the differential metabolite analysis. LC-MS data were processed using Progenesis QI default normalization, followed by missing-value filtering and imputation; QC-based LOESS signal-drift correction was applied to LC-MS data; LC-MS and GC-MS data matrices were separately batch-corrected using statTarget; study-sample injection order was randomized; pooled QC samples were inserted at regular intervals; and ion peaks with QC RSD >30% were removed before downstream analysis. In addition, the corrected QC RSD values of the eight pathway-mapped metabolites ranged from 1.45% to 3.64%, and the corrected internal-standard QC RSD values were 6.28% in negative-ion mode and 2.10% in positive-ion mode, supporting acceptable analytical reproducibility for the reported features. These feature-level QC data are provided in **Supplementary Table 9**.

However, the exact LC-MS normalization target implemented by the Progenesis QI default algorithm could not be specified at the manuscript level. L-2-chlorophenylalanine was used to monitor sample-preparation and instrumental stability but was not used for LC-MS signal normalization. Although solvent blanks were included during acquisition, blank raw data were not retained in the exported project record and were not used for background-peak or contaminant removal; therefore, blank-related plots or blank/sample ratio summaries could not be provided. Although sample-level intensity distribution plots before and after correction were provided for both GC-MS and LC-MS datasets (**Supplementary Figure 5**), the corrected LC-MS matrix contained a subset of very low log10-transformed intensity values derived mainly from KNN-imputed missing or below-detection-limit signals. These imputed values were retained in downstream analyses after preprocessing, correction, imputation, and QC filtering. Therefore, part of the corrected LC-MS intensity distribution was influenced by missing-value handling and should be considered when interpreting the differential metabolite findings. In addition, run-order drift plots for all retained features were not available from the exported project record. Finally, the reported LC-MS metabolites were putatively annotated rather than fully confirmed using authentic standards. Therefore, residual technical effects and uncertainty in metabolite identity cannot be completely excluded, and the metabolomics results should be interpreted as exploratory cross-sectional patterns under the present preprocessing workflow.

Moreover, the exploratory models in this study were developed as pairwise binary classifiers (CRC-m vs. CRC-nm and CRC-m vs. HC) and do not establish three-class discrimination across HC, CRC-nm, and CRC-m. ROC-AUC, sensitivity, specificity, PPV, NPV, F1-score, and PR-AUC were reported from an outer five-fold stratified cross-validation framework. For bacteria-only and integrated models, bacterial feature filtering was performed within each outer training fold, and the resulting training-fold-specific feature set was applied to the corresponding validation fold. In contrast, metabolite-only models and the metabolite component of integrated models used all detected serum metabolite features without additional univariate or differential-metabolite prescreening. Although this workflow reduced information leakage compared with full-dataset prescreening, the modeling analyses remain exploratory because of the limited sample size, absence of an independent external validation cohort, and complete stage confounding between CRC-m and CRC-nm. Therefore, the reported model performance should be interpreted as preliminary internal performance rather than evidence of clinical diagnostic utility. In addition, no formal statistical comparison between model performances was performed; therefore, differences in ROC-AUC or threshold-dependent metrics across model types should be interpreted descriptively.

Although dietary habits were collected from medical records and questionnaires, they were recorded only as a broad category (“mixed diet”) rather than by validated quantitative dietary assessment tools, which prevented meaningful dietary adjustment in the analysis. Given the important influence of diet on both gut microbiota and serum metabolite profiles, residual dietary confounding cannot be excluded. Future studies should include larger and multicenter cohorts, apply stage-stratified or multivariable analyses adjusting for demographic and clinical variables such as age, sex, BMI, tumor stage, primary tumor location, and CEA level, incorporate standardized dietary assessments, and perform prospective follow-up and functional experiments to further validate the stability and interpretability of these exploratory findings.

In conclusion, this study identified cross-sectional differences in gut microbiota composition, selected serum metabolites, inflammatory variables, and exploratory taxa–metabolite associations across HC, CRC-nm, and CRC-m/stage IV groups in the present cohort. Under the exploratory FDR-controlled LEfSe framework, *Enterocloster clostridioformis* and *Lactobacillus crispatus* were retained as shared CRC-m-enriched exploratory candidate species-level features, whereas *Megamonas rupellensis* and *Phocaeicola plebeius* were retained as shared CRC-m-depleted candidate features in the present cohort. Exploratory bacteria-only, metabolite-only, and integrated microbiota–metabolite models were evaluated using an outer five-fold stratified cross-validation framework, with performance reported using ROC-AUC, PR-AUC, sensitivity, specificity, PPV, NPV, and F1-score. In this exploratory workflow, adding bacterial features to metabolite-only models provided no incremental predictive benefit. Instead, integrated models showed lower internal performance for some model/comparison/metric combinations; notably, in the CRC-m versus CRC-nm comparison, integrated LASSO showed lower ROC-AUC, PR-AUC, sensitivity, NPV, and F1-score than metabolite-only LASSO, and integrated elastic net showed lower fixed-threshold performance for several metrics despite higher ROC-AUC/PR-AUC. Therefore, integrated microbiota–metabolite models should not be interpreted as equivalent or preferable to metabolite-only models. These modeling results should be interpreted descriptively rather than as statistically confirmed superiority or evidence of clinical diagnostic utility, especially because of the small sample size, the absence of independent external validation, and complete stage confounding between CRC-m and CRC-nm. Within-group correlation analyses showed only limited and group-specific taxa–metabolite associations after adjustment, and these results should be interpreted as exploratory rather than as evidence of direct functional microbiota–metabolite interactions. Because all CRC-m cases were stage IV and all CRC-nm cases were stages I–III, the observed microbial, metabolic, inflammatory, modeling, and correlation findings should not be interpreted as evidence of biology attributable specifically to metastatic dissemination, but rather as cross-sectional differences between clinically defined, stage-confounded groups that may reflect disease stage, tumor burden, current disease status, or broader progression-related changes. Overall, these findings provide exploratory cross-sectional microbial and metabolic candidate features for further validation in independent, stage-balanced cohorts rather than establishing metastasis-specific biomarkers. Future research should address population heterogeneity, stage-related confounding, residual confounding by demographic, clinical, tumor-related, and dietary factors, and mechanistic uncertainty to further evaluate the stability, reproducibility, and biological interpretability of these exploratory findings.

## Data Availability

The datasets presented in this study can be found in online repositories. The names of the repository/repositories and accession number(s) can be found in the article/**Supplementary Material**.

## Supplementary material

Supplementary Figure 1 Supervised PLS-DA score plots of serum metabolomics data. **(A)** PLS-DA score plot of the GC-MS dataset. **(B)** PLS-DA score plot of the LC-MS dataset. These plots are provided as supervised complementary visualizations of serum metabolomic differences across HC, CRC-nm, and CRC-m groups.

Supplementary Figure 2 Assessment of PLS-DA model overfitting. **(A, B)** Model quality was evaluated using 7-fold cross-validation and 200-times response permutation testing (RPT). Validity criteria: (1) all green Q2 values on the left are lower than the original points on the right; and (2) the green regression line for Q2 intersects the y-axis at or below zero. **(A)** GC-MS (all samples). **(B)** LC-MS (all samples).

Supplementary Figure 3 Quality-control assessment of the GC-MS data. **(A)** Total ion chromatogram (TIC) overlay of pooled quality-control (QC) samples, showing highly consistent retention-time profiles and signal response across injections. **(B)** Distribution of metabolite intensities across samples, including QC samples, demonstrating comparable overall signal distributions after preprocessing. **(C)** PCA score plot including QC samples, showing clustering of QC injections and supporting acceptable analytical reproducibility. **(D)** Hierarchical clustering heatmap including QC samples, showing that QC samples cluster closely and indicating satisfactory analytical stability during the run. The GC-MS data matrix was further processed using internal-standard normalization and statTarget-based batch correction, and features with QC RSD >30% were removed before downstream analysis.

Supplementary Figure 4 Quality-control assessment of the LC-MS data. **(A)** Distribution of metabolite intensities across samples, including QC samples, demonstrating comparable overall signal distributions after preprocessing. **(B)** PCA score plot including QC samples, showing clustering of QC injections and supporting acceptable analytical reproducibility. **(C)** Hierarchical clustering heatmap including QC samples, showing that QC samples cluster closely and indicating satisfactory analytical stability during the run. The LC-MS data matrix was processed using Progenesis QI default normalization, QC-based LOESS signal-drift correction, statTarget-based batch correction, and removal of features with QC RSD >30% before downstream analysis.

Supplementary Figure 5 Sample-level metabolite intensity distributions before and after correction for GC-MS and LC-MS datasets. **(A, B)** Sample-level log10-transformed metabolite intensity distributions before correction **(A)** and after correction **(B)** for the GC-MS dataset. **(C, D)** Sample-level log10-transformed metabolite intensity distributions for the LC-MS dataset. For LC-MS, panel **(C)** represents the Progenesis QI-normalized data after log10 transformation, before QC-based LOESS signal-drift correction, statTarget batch correction, and missing-value imputation. Panel **(D)** represents the corresponding matrix after QC-based LOESS correction, statTarget batch correction, and KNN-based missing-value imputation. The same retained LC-MS feature set is shown in panels **(C, D)**, but at different preprocessing stages. Each box represents the distribution of detected metabolite feature intensities within one sample. Samples are grouped as pooled quality-control samples (QC), healthy controls (HC), non-metastatic colorectal cancer (CRC-nm), and metastatic colorectal cancer (CRC-m). The dense low-intensity component around log10 values of approximately −6 to −8 in panel D mainly reflects small positive values generated during KNN-based imputation for features with original signals below the detection limit or absent in some samples. These imputed values were retained in the downstream differential metabolite analysis after preprocessing, correction, imputation, and QC filtering.
