# Supplementary figures and images for: Cross-sectional gut microbiota and serum metabolite differences across clinically defined groups in colorectal cancer
Source: Front Cell Infect Microbiol. 2026 Jul 10;16:1815707. doi: 10.3389/fcimb.2026.1815707 (PMC13395675; doi:10.3389/fcimb.2026.1815707)

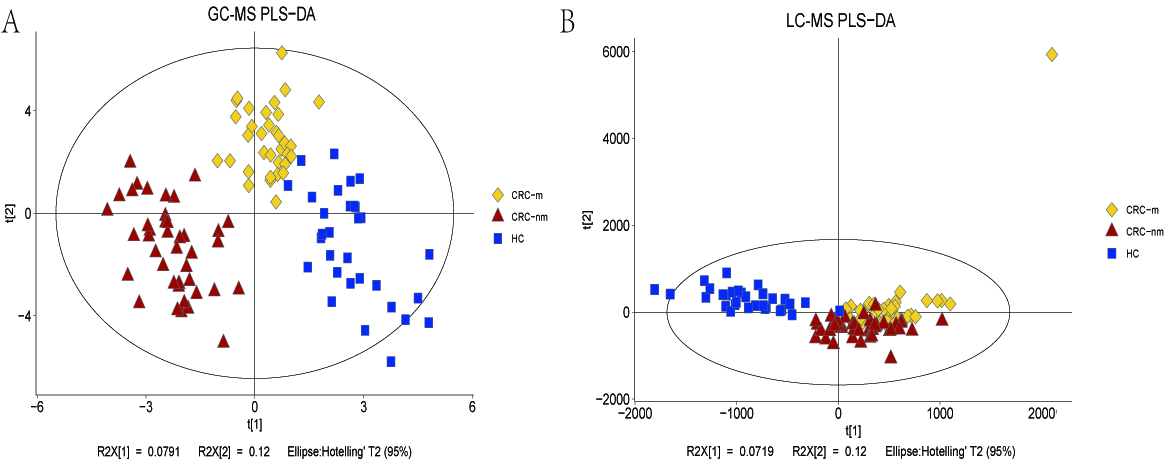

Supplement: Supplementary Figure 1 — Supervised PLS-DA score plots of serum metabolomics data. (A) PLS-DA score plot of the GC-MS dataset. (B) PLS-DA score plot of the LC-MS dataset. These plots are provided as supervised complementary visualizations of serum metabolomic differences across HC, CRC-nm, and CRC-m groups. [file Image1.tif]

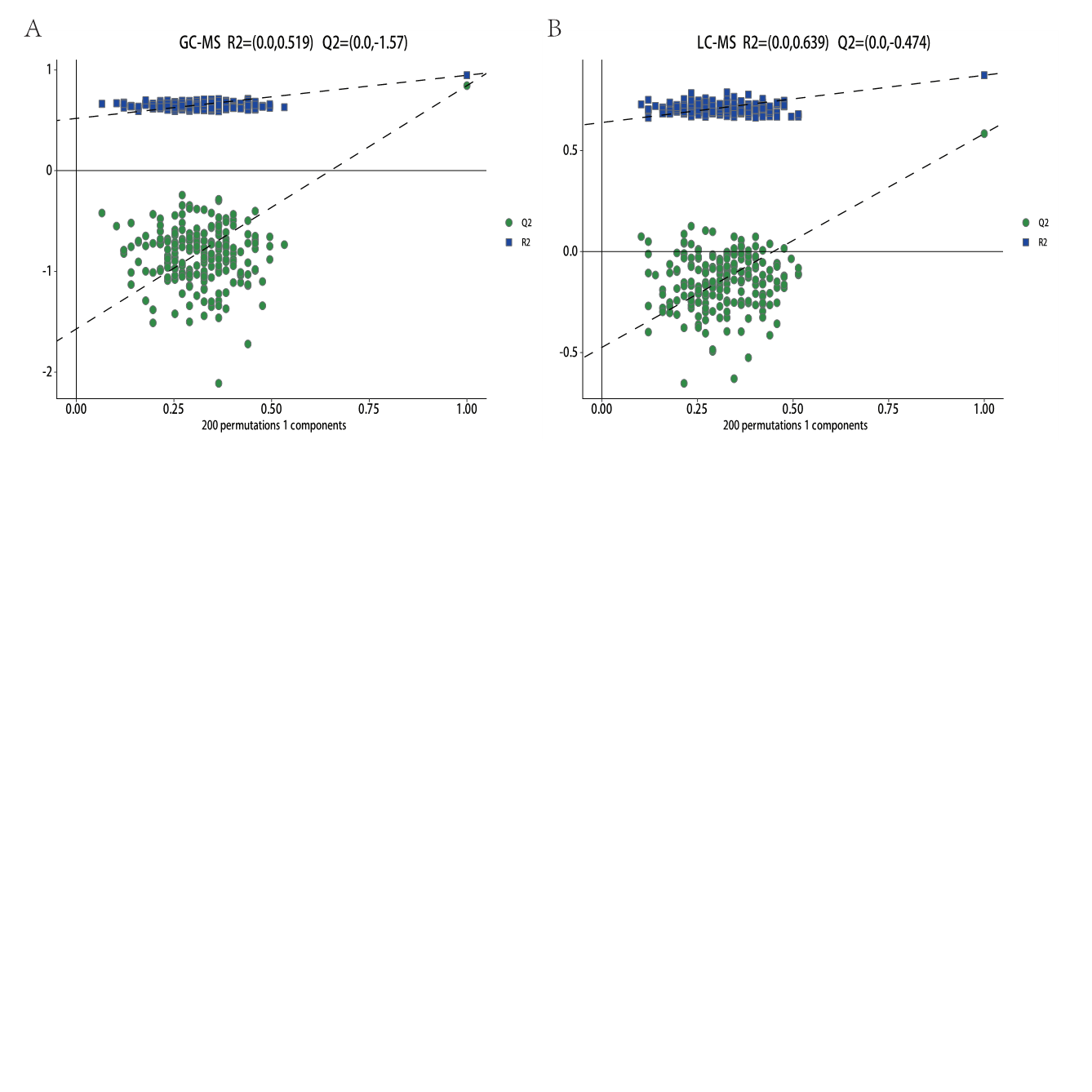

Supplement: Supplementary Figure 2 — Assessment of PLS-DA model overfitting. (A, B) Model quality was evaluated using 7-fold cross-validation and 200-times response permutation testing (RPT). Validity criteria: (1) all green Q2 values on the left are lower than the original points on the right; and (2) the green regression line for Q2 intersects the y-axis at or below zero. (A) GC-MS (all samples). (B) LC-MS (all samples). [file Image2.tiff]

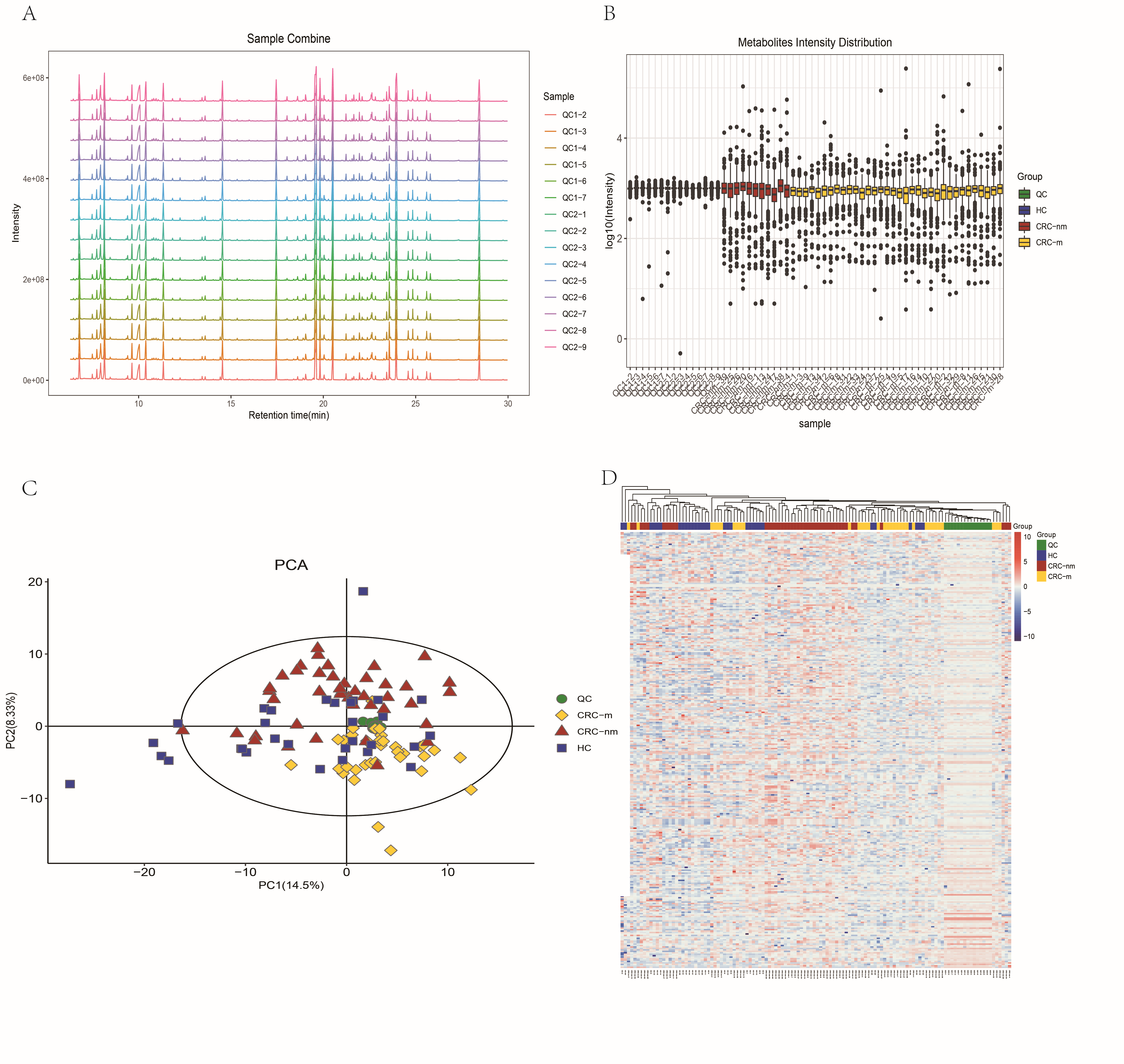

Supplement: Supplementary Figure 3 — Quality-control assessment of the GC-MS data. (A) Total ion chromatogram (TIC) overlay of pooled quality-control (QC) samples, showing highly consistent retention-time profiles and signal response across injections. (B) Distribution of metabolite intensities across samples, including QC samples, demonstrating comparable overall signal distributions after preprocessing. (C) PCA score plot including QC samples, showing clustering of QC injections and supporting acceptable analytical reproducibility. (D) Hierarchical clustering heatmap including QC samples, showing that QC samples cluster closely and indicating satisfactory analytical stability during the run. The GC-MS data matrix was further processed using internal-standard normalization and statTarget-based batch correction, and features with QC RSD >30% were removed before downstream analysis. [file Image3.tif]

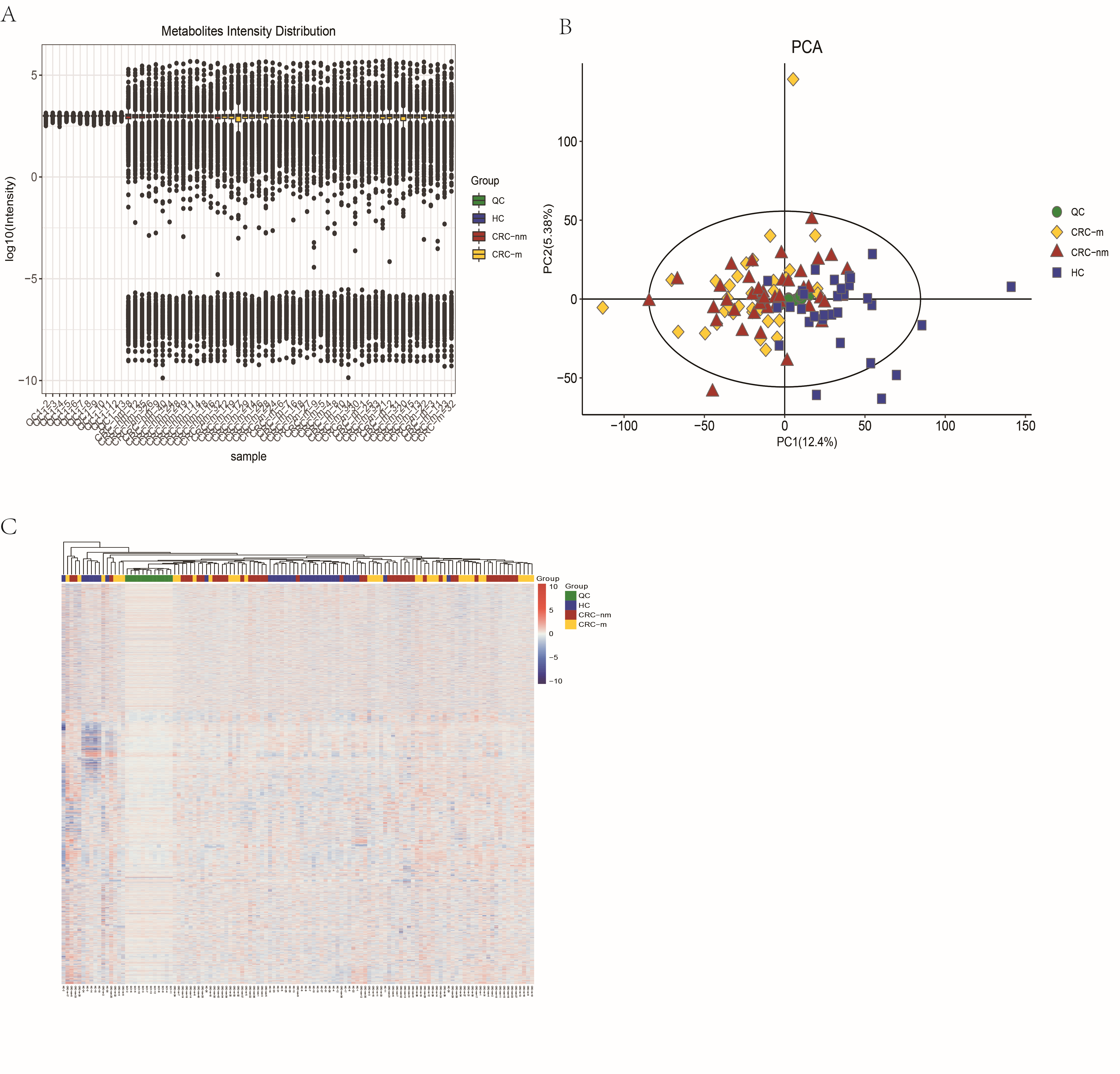

Supplement: Supplementary Figure 4 — Quality-control assessment of the LC-MS data. (A) Distribution of metabolite intensities across samples, including QC samples, demonstrating comparable overall signal distributions after preprocessing. (B) PCA score plot including QC samples, showing clustering of QC injections and supporting acceptable analytical reproducibility. (C) Hierarchical clustering heatmap including QC samples, showing that QC samples cluster closely and indicating satisfactory analytical stability during the run. The LC-MS data matrix was processed using Progenesis QI default normalization, QC-based LOESS signal-drift correction, statTarget-based batch correction, and removal of features with QC RSD >30% before downstream analysis. [file Image4.tif]

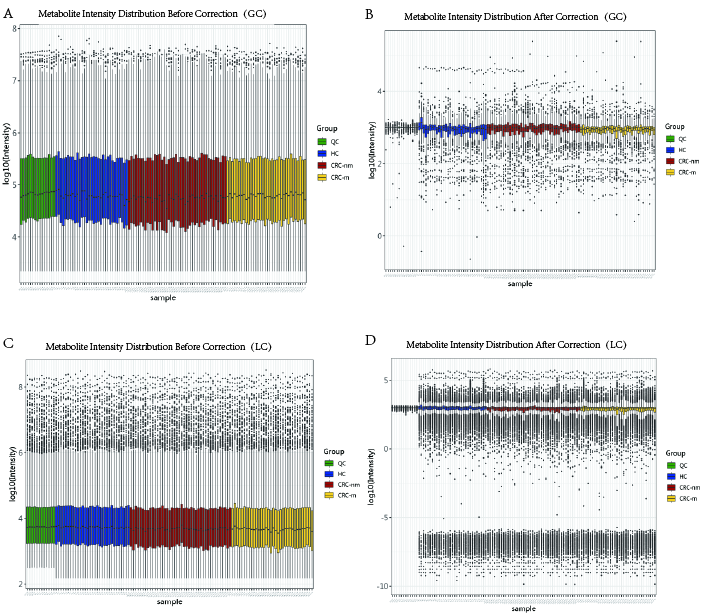

Supplement: Supplementary Figure 5 — Sample-level metabolite intensity distributions before and after correction for GC-MS and LC-MS datasets. (A, B) Sample-level log10-transformed metabolite intensity distributions before correction (A) and after correction (B) for the GC-MS dataset. (C, D) Sample-level log10-transformed metabolite intensity distributions for the LC-MS dataset. For LC-MS, panel (C) represents the Progenesis QI-normalized data after log10 transformation, before QC-based LOESS signal-drift correction, statTarget batch correction, and missing-value imputation. Panel (D) represents the corresponding matrix after QC-based LOESS correction, statTarget batch correction, and KNN-based missing-value imputation. The same retained LC-MS feature set is shown in panels (C, D), but at different preprocessing stages. Each box represents the distribution of detected metabolite feature intensities within one sample. Samples are grouped as pooled quality-control samples (QC), healthy controls (HC), non-metastatic colorectal cancer (CRC-nm), and metastatic colorectal cancer (CRC-m). The dense low-intensity component around log10 values of approximately −6 to −8 in panel D mainly reflects small positive values generated during KNN-based imputation for features with original signals below the detection limit or absent in some samples. These imputed values were retained in the downstream differential metabolite analysis after preprocessing, correction, imputation, and QC filtering. [file Image5.tif]
